# Supplementary material for: Gain of CTCF-Anchored Chromatin Loops Marks the Exit from Naive Pluripotency
Source: Cell Syst. 2018 Nov 28;7(5):482–495.e10. doi: 10.1016/j.cels.2018.09.003 (PMC6327227; doi:10.1016/j.cels.2018.09.003)
Supplement: Document S1. Figures S1–S7 and Table S1 [file mmc1.pdf]

Cell Systems, Volume 7

Supplemental Information

Gain of CTCF-Anchored Chromatin Loops

Marks the Exit from Naive Pluripotency

Aleksandra Pękowska, Bernd Klaus, Wanqing Xiang, Jacqueline Severino, Nathalie Daigle, Felix A. Klein, Małgorzata Oleś, Rafael Casellas, Jan Ellenberg, Lars M. Steinmetz, Paul Bertone, and Wolfgang Huber

Supplementary Table 1, related to Figure 1 | Chromatin conformation data produced for this study.

Sample	Protocol	Number of sequenced reads	Uniquely aligned read pairs	Final contacts (MAPQ≥30)	Intra-chromosomal (%)	Inter-chromosomal (%)
**ESC (FBS/LIF) rep1**	TCC	134,434,660	81,290,648	53,023,831	89.1	10.85
**ESC (FBS/LIF) rep2**	TCC	171,193,881	105,990,730	69,261,153	89.3	10.6
**NSC rep1**	TCC	127,309,593	94,140,986	64,491,743	89.6	10.4
**NSC rep2**	TCC	171,338,684	85,238,722	49,202,635	87.2	12.8
**ESC (2i/LIF) rep1**	TCC	120,729,477	93,663,961	62,710,291	90.1	9.9
**ESC (2i/LIF) rep2**	TCC	146,159,495	110,767,469	71,398,936	88.2	11.8
**EpiSC rep1**	TCC	72,946,843	47,857,076	32,824,806	85.7	14.3
**EpiSC rep2**	TCC	266,948,104	177,889,434	113,867,789	87.8	12.2
**ESC (FBS/LIF) rep1**	*In situ* Hi-C	611,626,283	561,290,686	399,655,481	87.6	12.4
**ESC (FBS/LIF) rep2**	*In situ* Hi-C	622,966,917	572,610,548	408,970,163	83.5	16.5
**NSC rep1**	*In situ* Hi-C	657,535,758	604,637,569	430,767,343	81.3	18.7
**NSC rep2**	*In situ* Hi-C	619,104,144	563,686,394	398,857,642	80.9	19.1

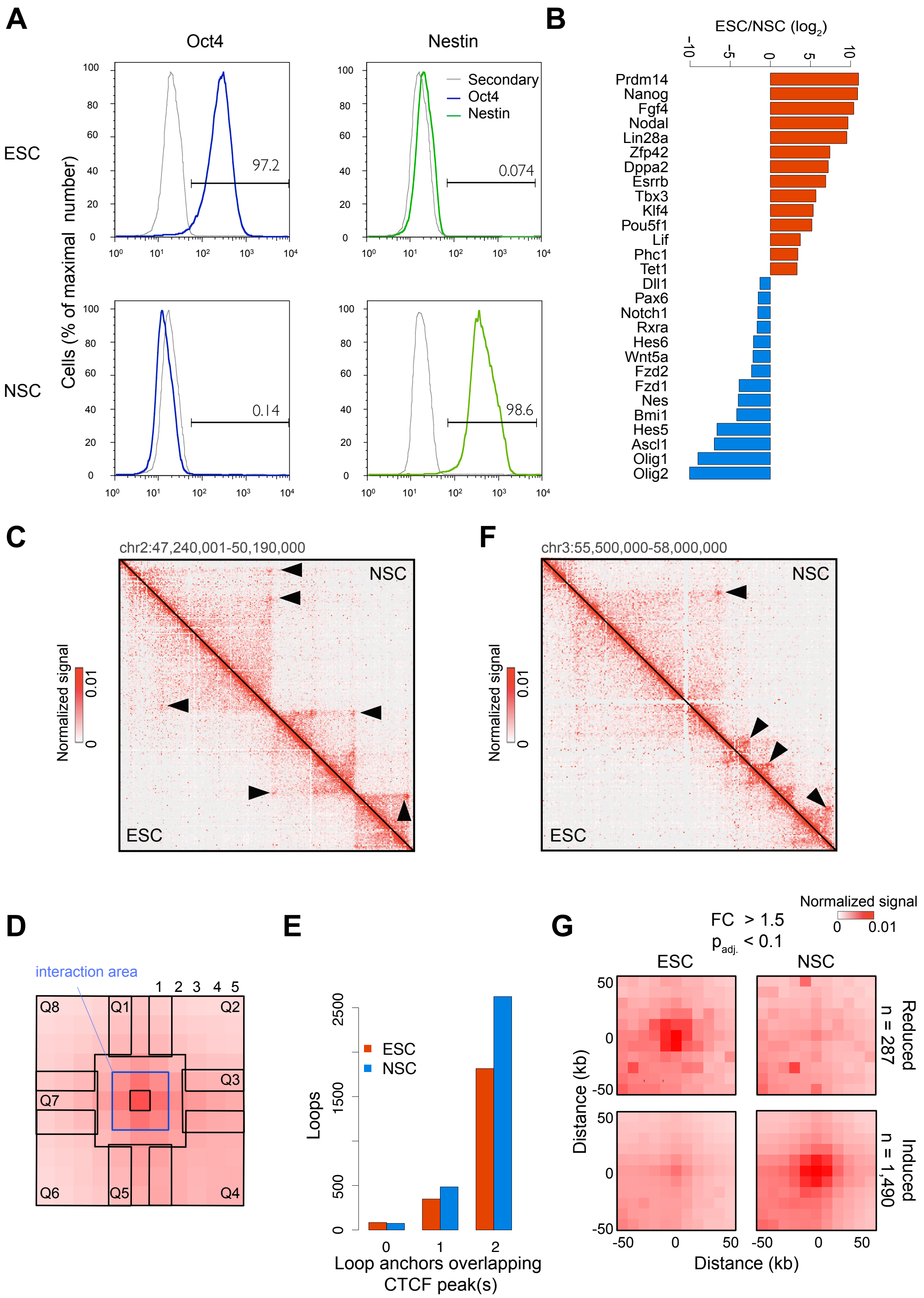

Figure S1, related to Figure 1 | Loops are primarily gained upon differentiation. A) Flow cytometry analysis of Oct4 and Nestin in ESCs and NSCs. B) Comparative expression of key pluripotency (red) and neural stem cell-related genes (blue) from RNA-seq data (adjusted p<0.0001, *DESeq* method). C) Example of chromatin loops in ESCs and NSCs (TCC data). D) Approach for the identification of loops. Bin pairs (pixels) in the area -50 to + 50 kb around each interacting 10 kb bin pair were analyzed for the presence of interactions. Eight overlapping areas of varying shape were considered (Q1 to Q8, green and white areas in the schematic; note that some of these overlap others). The number of significant interactions in the pixels immediately surrounding and including the center pixel (interaction area) was counted. Loops were defined as pixels where this number was at least two in the interaction area, and where at most two interactions were observed in each of areas Q1 to Q8. E) The majority of loops identified from TCC data connect anchors overlapping CTCF binding sites. F) Example of loops induced in NSCs. G) Composite profile of normalized TCC signal from reduced (top) and induced (bottom) loops in ESCs (left) and NSCs (right) cells (Wald test, adjusted p=0.1 and FC>1.5).

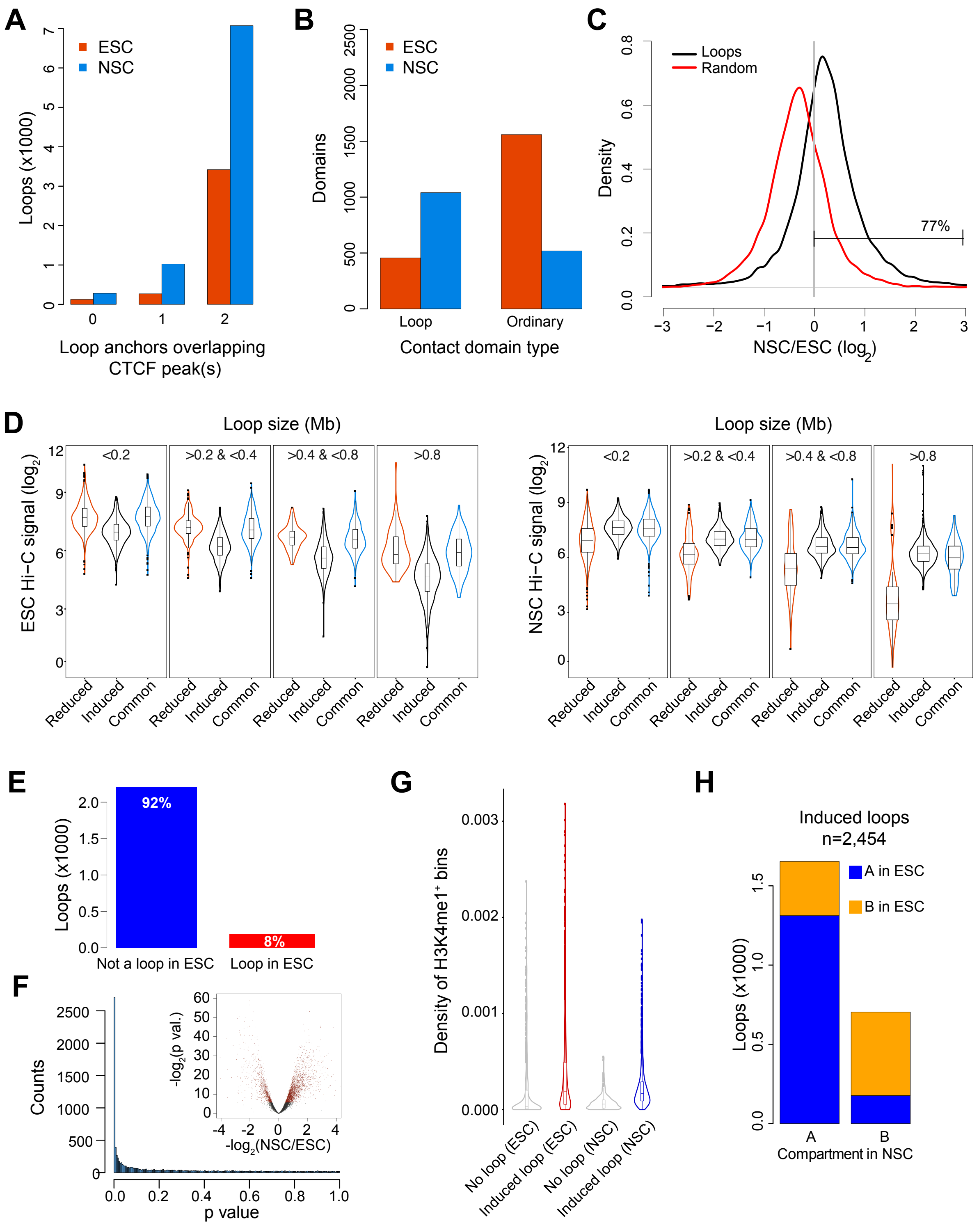

Figure S2, related to Figure 1 and Figure 2 | High resolution in-situ Hi-C reveals gain of loops upon differentiation. A) Loops increase upon ESC differentiation to NSCs. The majority of loops connect loci overlapping CTCF binding sites. B) The ratio of loops to ordinary domains increases upon differentiation. C) Loop signal is primarily increased over random (*in situ* Hi-C data). Normalized loop signal in ESCs and NSCs was considered (STAR Methods). D) Loops defined as dynamic via *DESeq2*-based analysis (Wald test) display signals comparable to common loops in the condition where they are identified as significantly stronger (*in situ* Hi-C data). E) Loops induced upon differentiation are largely undetectable in ESC. Only 8% of loops displaying signals stronger in NSCs (Wald test, FDR=0.05 and FC>1.5) are identified in ESCs (*HiCUPPS* method). F) Distribution of p-values from comparison of loop signals in ESC and NSC (Wald test, *in situ* Hi-C data). Inset: volcano plot depicting the relationship between $\log_{10}$ p values to $\log_2$ fold change of loop signals. G) Loops are formed in regions enriched for H3K4me1. Induced loop domains are already established in open chromatin in ESCs. H) Genomic intervals defined by the anchors of loops induced upon ESC differentiation to NSCs are within the euchromatic compartment (A) in both cell types. Compartmental assignment was evaluated genome-wide at 50 kb resolution. Loops were assigned to compartments based on annotation of the majority of bins mapping within the loop interval.

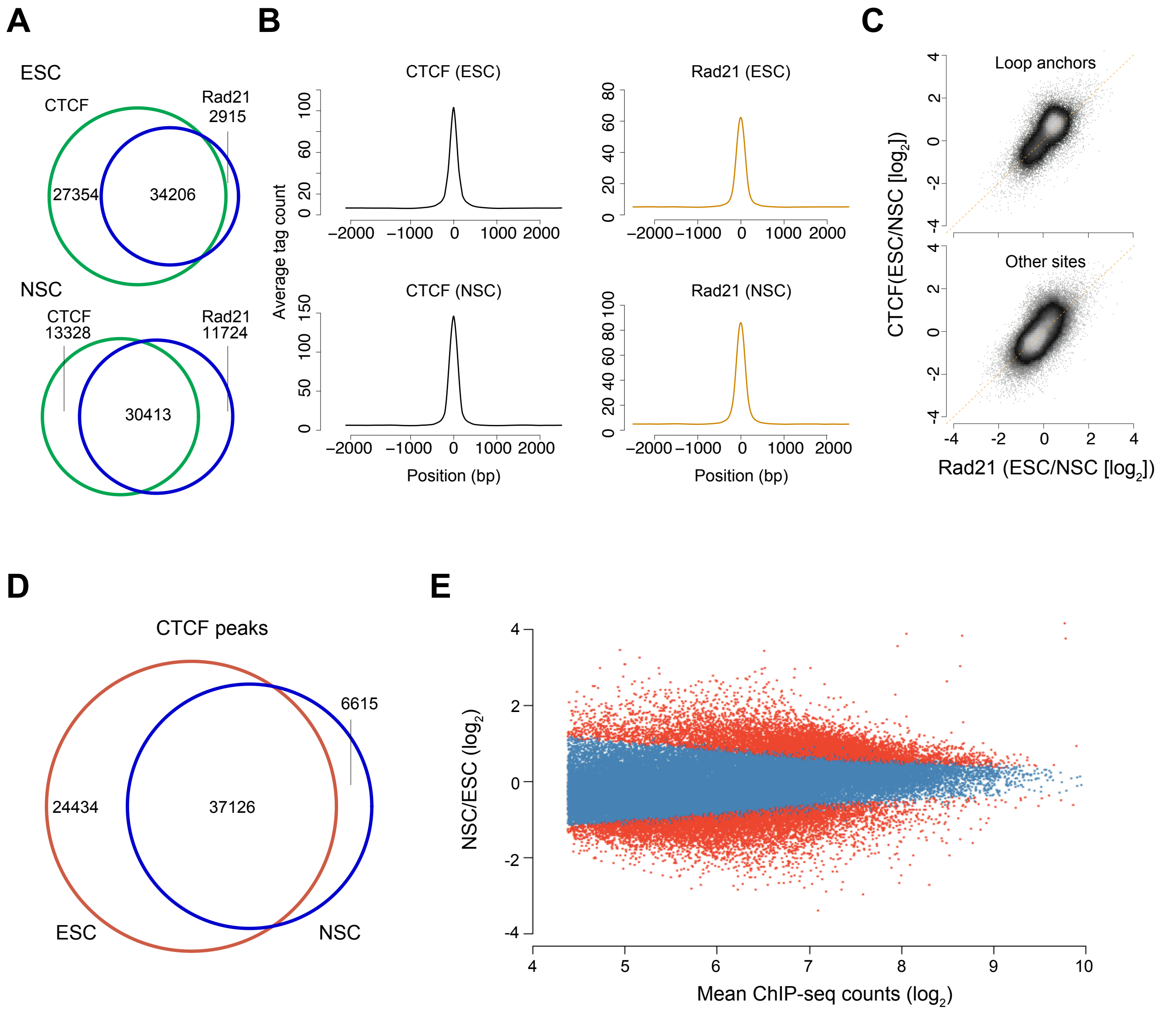

Figure S3, related to Figure 3 | CTCF and Rad21 following ESC differentiation to NSC. A) Overlap between the genomic positions of CTCF and Rad21 peaks identified in ESCs (top) or NSCs (bottom). B) Average profile of CTCF and Rad21 ChIP-seq fragments around the summits of CTCF peaks in ESCs and NSCs (top and bottom panels, respectively). C) Variation in CTCF and Rad21 signals in ESCs and NSCs is highly correlated both at loop anchors (*in situ* Hi-C data) and at other CTCF sites throughout the genome. *DESeq2*-normalized ChIP-seq signals in ESCs and NSCs were considered. D) Comparison of CTCF peaks identified in ESCs and NSCs. E) Log_2 fold change (NSC/ESC) of CTCF ChIP-seq signal in the function of the average signal strength (MA plot). Red: peaks with significant changes in CTCF binding (adjusted $p<0.1$).

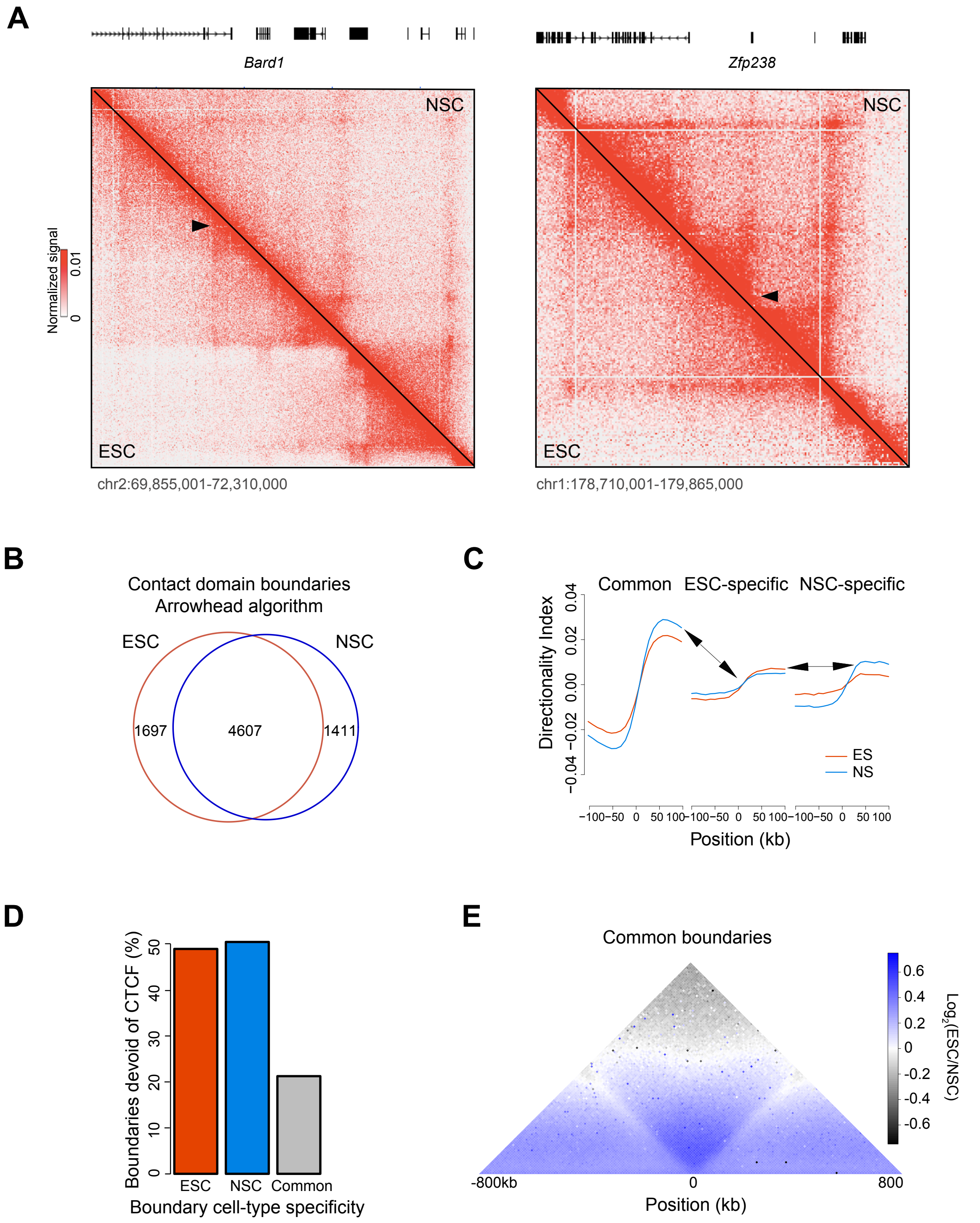

Figure S4, related to Figure 4 | Contact domain boundaries are weaker in ESC than in NSC. A) Cell type-specific contact domain boundaries. Left: ESC-specific contact domain boundary at the 3' end of the transcriptionally down-regulated *Bard1* locus (adjusted p<0.1, log_2(ESC/NSC)>1.5, *DESeq* method). Right: NSC-specific contact domain boundary at the 5' end of transcriptionally upregulated *Zfp238* (adjusted p<0.1, log_2(ESC/NSC)<-1.5, *DESeq* method). B) The majority of boundaries are common to both cell types. Shared and unique domain boundaries identified in ESCs and NSCs. C) Average profiles of the directionality index around common, ESC- and NSC-specific contact domain boundaries. Cell type-specific boundaries are weaker than shared instances. Overall, boundaries are stronger in NSCs than in ESCs. D) Cell type-specific boundaries are more frequently devoid of CTCF than common ones. E) Composite profile of normalized *in situ* Hi-C contacts around common boundaries of contact domains. ESCs display elevated numbers of Hi-C contacts crossing common domain boundaries.

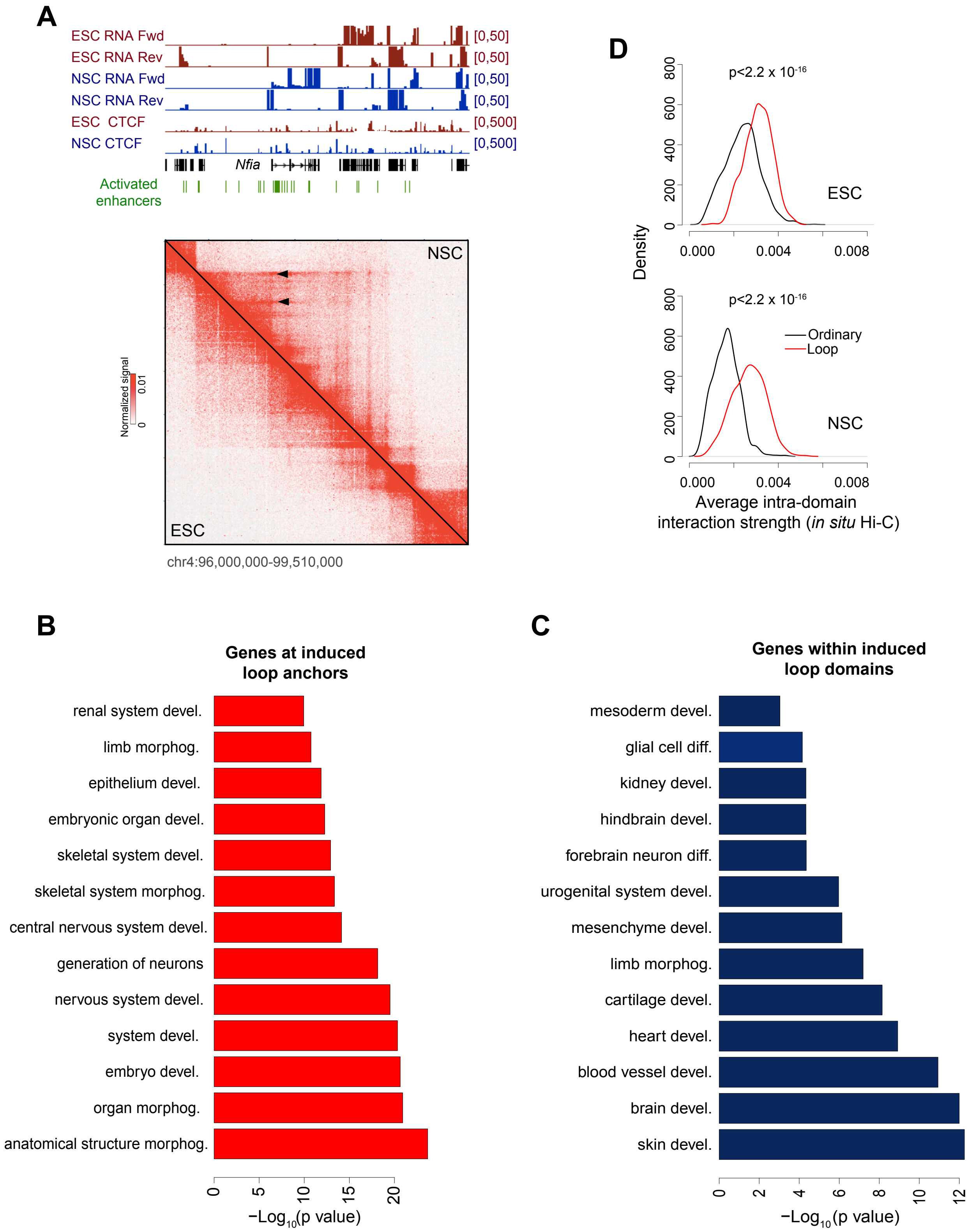

Figure S5, related to Figure 6 | Loop induction is associated with developmental control of gene expression. A) Induction of loops connecting the promoter of *Nfia* with distal regulatory elements in NSCs compared to ESCs. B) Gene Ontology (GO) analysis of genes with promoter regions intersecting anchors of induced loops. GO terms associated with developmental processes are displayed (Benjamini-Hochberg corrected p values). C) Genes within genomic intervals defined by the anchors of induced loops are implicated in development. Analysis as in panel B. D) Loop domains display stronger interactions on average than ordinary contact domains (two-sided t test). Interactions between loci located closer than 20kb were not considered in this analysis.

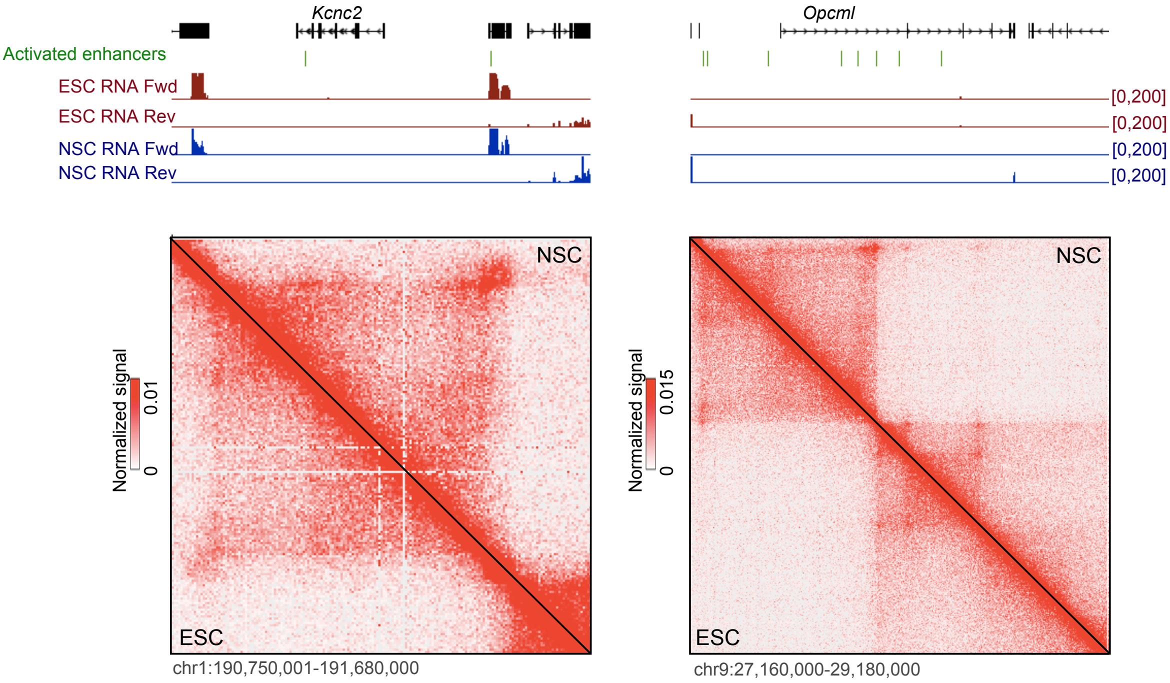

Figure S6, related to Figure 6 | Loops around genes expressed in adult neuronal tissues are induced in NSCs. Induced loops around *Kcnc2* (right), and *Opcml* (left), neural genes not yet induced in NSCs (*in situ* Hi-C data).

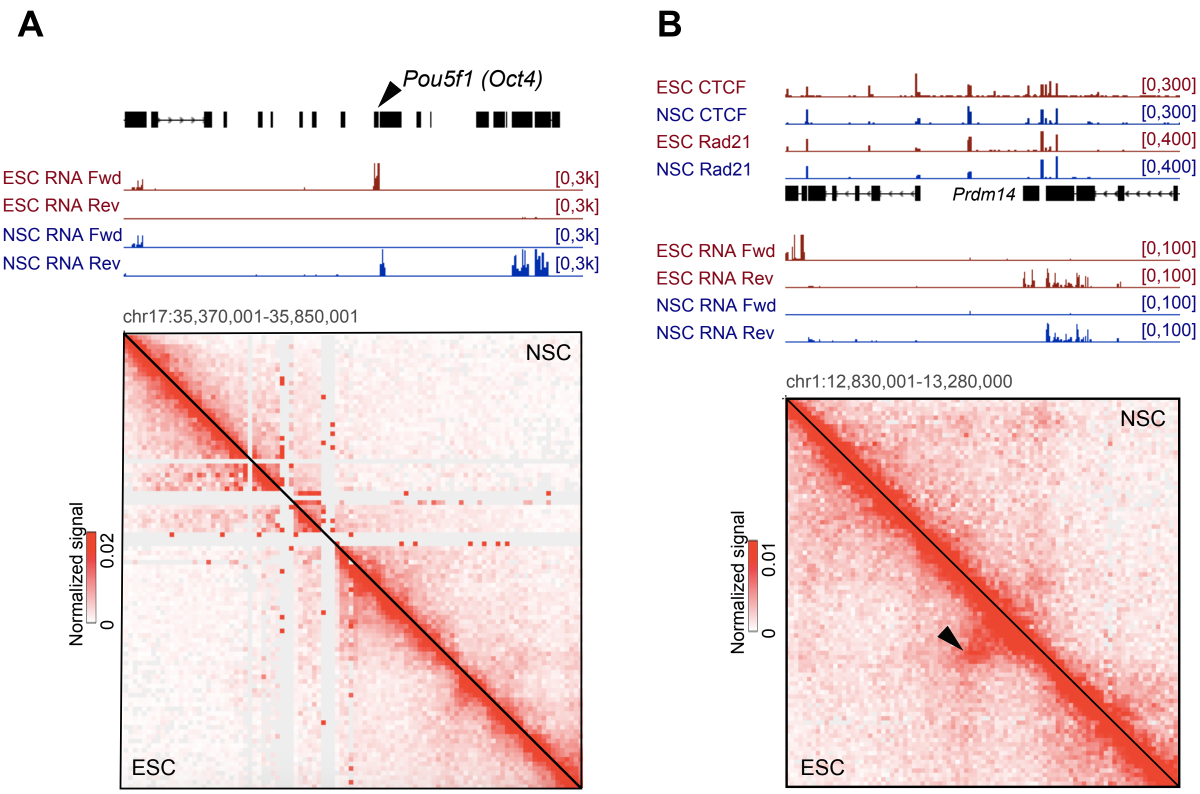

Figure S7, related to Figure 6 | Loop dynamics around pluripotency related genes. A) Absence of long-range loops around the *Pou5f1* (*Oct4*) locus in ESCs and NSCs. B) Short-range loop involving the promoter of pluripotency-associated gene *Prdm14*. This structure is present in ESCs and significantly diminished in NSCs ($p<10^{-4}$, $\log_2$(NSC/ESC) = -1.16). *Prdm14* is downregulated upon exit from pluripotency ($p<10^{-10}$, *DESeq* method, Figure S1B).
