# Supplementary material for: Gain of CTCF-Anchored Chromatin Loops Marks the Exit from Naive Pluripotency
Source: Cell Syst. 2018 Nov 28;7(5):482–495.e10. doi: 10.1016/j.cels.2018.09.003 (PMC6327227; doi:10.1016/j.cels.2018.09.003)
Supplement: Document S2. Article plus Supplemental Information [file mmc2.pdf]

Cell Systems

Article

Gain of CTCF-Anchored Chromatin Loops Marks the Exit from Naive Pluripotency

Graphical Abstract

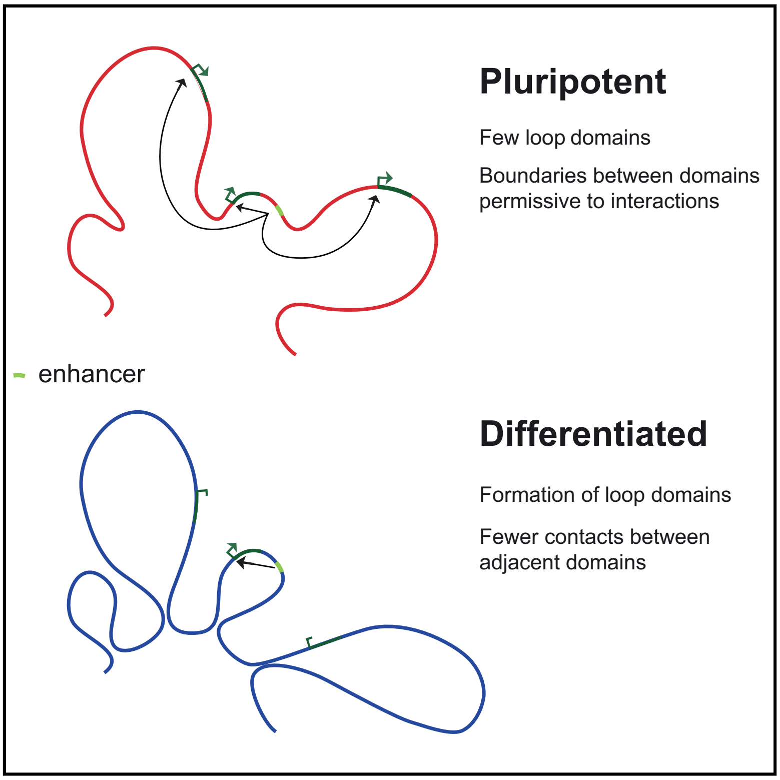

Highlights

- Genome-wide induction of CTCF-anchored loops accompanies loss of pluripotency
- Established chromatin domain boundaries are reinforced upon cell differentiation
- Newly formed long-range loop structures rarely connect enhancers and promoters
- Induced loop domains often encompass genes regulating developmental processes

Authors

Aleksandra Pękowska, Bernd Klaus, Wanqing Xiang, ..., Lars M. Steinmetz, Paul Bertone, Wolfgang Huber

Correspondence

aleksandra.pekowska@embl.de (A.P.), bertone@stemcells.cam.ac.uk (P.B.), whuber@embl.de (W.H.)

In Brief

We show that chromatin loops are infrequent and primarily short range in pluripotent stem cells. Differentiation to multipotent neural precursors induces genome-wide formation of loop structures and reinforcement of boundaries between chromatin interaction domains. Induced loops frequently arise around genes encoding developmental regulators. Reprogramming somatic cells to pluripotency reverts these phenomena.

Pękowska et al., 2018, Cell Systems 7, 482–495
November 28, 2018 Published by Elsevier Inc.
https://doi.org/10.1016/j.cels.2018.09.003

OPEN ACCESS
CellPress

Gain of CTCF-Anchored Chromatin Loops Marks the Exit from Naive Pluripotency

Aleksandra Pękowska,[1,*] Bernd Klaus,[1,9] Wanqing Xiang,[4,9] Jacqueline Severino,[1,9] Nathalie Daigle,[2] Felix A. Klein,[1] Małgorzata Oleś,[1] Rafael Casellas,[2,3] Jan Ellenberg,[4] Lars M. Steinmetz,[1,5,6] Paul Bertone,[1,7,8,*] and Wolfgang Huber[1,10,*]
[1]European Molecular Biology Laboratory (EMBL), Genome Biology Unit, Meyerhofstrasse 1, Heidelberg 69117, Germany
[2]Genomics & Immunity, National Institute of Arthritis and Musculoskeletal and Skin Diseases, National Institutes of Health, Bethesda, MD 20892, USA
[3]Center for Cancer Research, NCI, National Institutes of Health, Bethesda, MD 20892, USA
[4]European Molecular Biology Laboratory (EMBL), Cell Biology and Biophysics Unit, Meyerhofstrasse 1, Heidelberg 69117, Germany
[5]Stanford Genome Technology Center, 855 California Ave, Palo Alto, CA 94305, USA
[6]Department of Genetics, Stanford University School of Medicine, Stanford, CA 94305, USA
[7]European Molecular Biology Laboratory (EMBL), European Bioinformatics Institute, Wellcome Trust Genome Campus, Cambridge CB10 1SD, UK
[8]Wellcome Trust - Medical Research Council Stem Cell Institute, University of Cambridge, Tennis Court Road, Cambridge CB2 1QR, UK
[9]These authors contributed equally
[10]Lead Contact
*Correspondence: aleksandra.pekowska@embl.de (A.P.), bertone@stemcells.cam.ac.uk (P.B.), whuber@embl.de (W.H.)
https://doi.org/10.1016/j.cels.2018.09.003

SUMMARY

The genome of pluripotent stem cells adopts a unique three-dimensional architecture featuring weakly condensed heterochromatin and large nucleosome-free regions. Yet, it is unknown whether structural loops and contact domains display characteristics that distinguish embryonic stem cells (ESCs) from differentiated cell types. We used genome-wide chromosome conformation capture and super-resolution imaging to determine nuclear organization in mouse ESC and neural stem cell (NSC) derivatives. We found that loss of pluripotency is accompanied by widespread gain of structural loops. This general architectural change correlates with enhanced binding of CTCF and cohesins and more pronounced insulation of contacts across chromatin boundaries in lineage-committed cells. Reprogramming NSCs to pluripotency restores the unique features of ESC domain topology. Domains defined by the anchors of loops established upon differentiation are enriched for developmental genes. Chromatin loop formation is a pervasive structural alteration to the genome that accompanies exit from pluripotency and delineates the spatial segregation of developmentally regulated genes.

INTRODUCTION

Three-dimensional chromatin topology is an integral facet of transcriptional regulation in development and disease (Bickmore and van Steensel, 2013). Physical proximity of regulatory elements facilitates interactions between promoters and enhancers (Deng et al., 2014). Despite this, cognate enhancer-promoter pairs are frequently separated by vast genomic distances (Kieffer-Kwon et al., 2013; Sanyal et al., 2012; Spitz, 2016).

Megabase-sized regions of self-association, termed topologically associated domains (TADs) (Dixon et al., 2012; Nora et al., 2012; Sexton et al., 2012), provide a framework for understanding how contacts between *cis*-regulatory elements are orchestrated. TADs encompass clusters of cognate regulatory elements (Sanyal et al., 2012; Symmons et al., 2014; Tsujimura et al., 2015) and mediate efficient contacts within domains (Symmons et al., 2016). Likewise, expression patterns of genes encompassed within a TAD are significantly correlated (Shen et al., 2012; Zhan et al., 2017). TAD boundaries are enriched in CTCF binding sites (Dixon et al., 2012; Rao et al., 2014) and function as insulators by blocking ectopic enhancer-promoter transactions between adjacent domains (Flavahan et al., 2016; Lupiáñez et al., 2015). High-resolution chromatin conformation maps have revealed fine structures of TADs (Phillips-Cremins et al., 2013), which are composed of smaller, frequently nested contact domains (Rao et al., 2014).

The formation of a chromatin domain is frequently accompanied by a structural loop (Rao et al., 2014). Structural loops manifest in chromatin conformation data as focal interaction points (Rao et al., 2014). Boundaries of approximately 40% of chromatin domains are connected by a loop (termed loop domains; the remaining chromatin domains are referred to as ordinary domains). CTCF has emerged as the central factor underlying loop formation, as 85% of loops bridge CTCF-bound loci, which are primarily facing each other (i.e., convergent; Rao et al., 2014; Vietri Rudan et al., 2015; de Wit et al., 2015). Reductions in CTCF occupancy result in concomitant depletion of loop structures (Nora et al., 2017).

Most studies relating nuclear topology to development have focused on the analysis of TADs. These structures are detected from the 8-cell stage of mouse embryogenesis (Du et al., 2017; Ke et al., 2017). Although high-resolution chromatin conformation maps show that cell differentiation alters the internal structure of TADs (Dixon et al., 2015; Phillips-Cremins et al., 2013),

This is an open access article under the CC BY license (http://creativecommons.org/licenses/by/4.0/).

it remains unclear whether and how specific TAD structures are related to lineage specialization. For example, embryonic stem cells (ESCs) feature large nucleosome-free regions (Ricci et al., 2015) and widespread transcriptional activity (Efroni et al., 2008; Marks et al., 2012). Consequently, chromatin conformation capture using locus-directed (4C) or global high-throughput sequencing (Hi-C) methods revealed that transcriptionally inactive regions engage in long-range contacts at lower frequency in ESCs than in differentiated cells (de Wit et al., 2013). Exceptions to this general rule are loci bound by core pluripotency regulators, which form large-scale contacts specific to ESCs (Apostolou et al., 2013; Denholtz et al., 2013; Wei et al., 2013; de Wit et al., 2013).

Despite marked features of chromatin arrangement associated with pluripotency, domain boundaries seem largely unaffected by ESC differentiation, and TAD boundaries are conserved in diverse cell types across developmental stages (Dixon et al., 2012; Rao et al., 2014). These observations raise questions pertaining to the relationship between the insulatory strength of contact domain boundaries and the plasticity of ESCs. Given that chromatin domain boundaries are frequently connected by loops, quantitative analysis of loop strength during cell differentiation will be instrumental to characterize the interplay between changes in contact domain architecture and developmental progression. In particular, it remains unclear how the formation and dissolution of higher-order chromatin structures take place in relation to cell-state transitions.

Here, we dissect the relationship between chromatin loop formation, contact domain architecture, and changes in cell identity that accompanies differentiation and restriction of developmental potential. We show that pluripotent stem cells form fewer loops genome-wide than more specialized progeny. Following differentiation of mouse ESCs to neural stem cells (NSCs), however, loop strength is increased, suggesting that engagement in a developmental program triggers the establishment of a chromatin conformation state where preexisting structures are later reinforced in more mature cell types. Newly formed loop domains (1) displayed increased spatial separation from neighboring genomic loci, (2) contained enhancers activated upon differentiation, and (3) spanned genes associated with developmental processes.

Hence, at the level of nuclear organization, the specificity of *cis*-regulatory element contacts is enhanced in response to differentiation. Consequently, we find that ESCs are characterized by generally weaker contact domain boundaries than those present in specialized counterparts. We go on to show that the consolidation of boundaries is explained by a global increase in CTCF binding at these locations upon induction to the neural lineage. Aligning with recent literature (Bonev et al., 2017; Stadhouders et al., 2018), we conclude that lower prevalence of large-scale structural loops and weaker chromatin contact insulation are hallmarks of three-dimensional genome organization in pluripotent cells.

RESULTS

We induced mouse ESCs to the neural lineage and a multipotent NSC identity in a directed differentiation assay (Conti et al., 2005; Ying et al., 2003). We assessed marker presentation of each population by flow cytometry and transcriptional state by high-throughput sequencing (RNA sequencing [RNA-seq]) (Figures S1A and S1B). We then analyzed chromatin conformation in the two cell types. To compare structural loops between ESCs and NSCs, we produced tethered chromosome conformation capture (TCC) (Kalhor et al., 2011) libraries from both cell types (Table S1). We identified a total of 4,328 chromatin loops in ESCs, NSCs, or both at a resolution of 10 kb (Figures S1C and S1D; STAR Methods). We profiled DNA association of CTCF by chromatin immunoprecipitation followed by high-throughput sequencing (chromatin immunoprecipitation sequencing [ChIP-seq]) and assessed the overlap between loop anchors and CTCF binding. In both ESCs and NSCs, more than 85% of loops connected two CTCF binding sites (Figure S1E). We observed substantially greater numbers of CTCF-anchored loops in differentiated NSCs than in parental ESCs (2,625 versus 1,815; Figures S1E and S1F). To assess variation in loop signal, we compared normalized read counts at loops using a statistical test for count data (Wald test; STAR Methods). We found a majority of 1,490 to display greater signal in NSCs, compared to 287 weaker instances (FC > 1.5; false discovery rate [FDR] = 0.1; Figure S1G). These observations indicate that chromatin loops are, as expected, generally associated with CTCF occupancy and that CTCF anchored loops are more prevalent in NSCs than in ESCs.

To substantiate these results, we generated a second dataset using a different conformation capture method. We produced high-resolution chromatin interaction maps by *in situ* Hi-C (Rao et al., 2014) on the same cell populations. We sequenced 2.5 billion reads and obtained a total of 1.6 billion high-quality Hi-C contacts (Table S1; STAR Methods). Using *juicer* (Durand et al., 2016a), we identified 3,817 and 8,382 loops in ESCs and NSCs, respectively (Figures 1A, S2A, and S2B). We considered the union of instances from both cell populations (n = 9,841) and observed an overall increase in loop signal upon establishment of NSC cultures (mean FC = 1.2; $p < 2.2 \times 10^{-16}$; two-sided t test; Figure S2C; for p values, we follow the convention used by the statistical software *R* to report values below 2.2×10^{-16} as $< 2.2 \times 10^{-16}$). Under stringent criteria (Wald test, FDR = 0.05, FC > 1.5), 2,454 loops were induced and 811 reduced (Figures 1B and 1C). Dynamic loops were found to be highly cell-type-specific (Figure S2D), and the overwhelming majority of induced loops (2,251 out of 2,454, i.e., 92%; Figures S2E and S2F) were below detection in ESCs. We then compared gained and lost loops across different ranges of genomic distance (Figure 1D). Long-range loops (>1.6 Mb) showed the most dramatic difference: in NSCs, they were present 18.4 times more often than absent (791 versus 43; $p < 2.2 \times 10^{-16}$; binomial test) in comparison to ESCs, and NSC-specific long-range loops were 8.6 times more abundant than those common to both cell types (FC < 1.25; n = 3,917). Therefore, we conclude that loss of pluripotency correlates with widespread induction of long-range loops.

Next, we investigated whether reduced chromatin looping in ESCs could be attributed to an overall lower physical compaction of chromatin in this cell type. We used super-resolution imaging (SRI) to quantify ultrastructure variations in chromatin, as embodied by rearrangements of replication forks. Because loops were most frequent in euchromatin for both ESC and

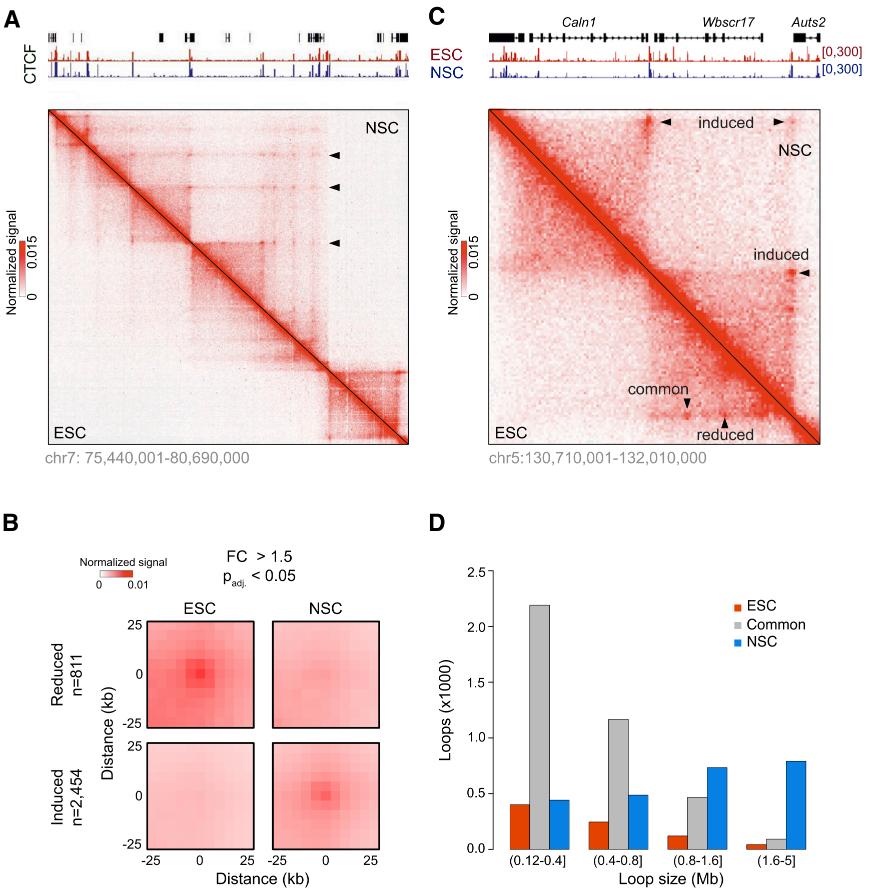

Figure 1. Differentiation Elicits Formation of Long-Range Chromatin Loops

(A) Examples of chromatin loops (arrows) in ESCs and NSCs (lower and upper triangles, respectively). Heatmaps show normalized counts of *in situ* Hi-C reads between pairs of genomic loci (STAR Methods).

(B) Composite profile of *in situ* Hi-C signal (similar to implementation of APA [Rao et al., 2014]) from reduced (top) and induced (bottom) loops in ESCs (left) and NSCs (right). Statistical significance of loop signal was assessed by a Wald test (FDR = 0.05 and FC > 1.5; STAR Methods).

(C) Examples of dynamic and stable loops.

(D) Length distributions of NSC-specific, common, and ESC-specific loops.

NSC (Figures S2G and S2H), we focused on early replicating domains (RDs), which tend to encompass transcriptionally active euchromatin. We labeled actively RDs (Xiang et al., 2018) in ESCs transformed with the FUCCI cell-cycle reporters (Roccio et al., 2013). We pulsed cells with EdU (Zessin et al., 2012), isolated those in early S-phase, and cultured the resulting population in either self-renewal or neural differentiation conditions for 96 hr (Figure 2A and STAR Methods). We measured the spatial arrangement of 2,410 RDs from 24 individual ESCs by SRI and of 2,576 RDs from 19 Nestin^{+} NSCs through nearest neighbor distance (NND) analysis (Figure 2B). Distributions of NNDs between individual RDs were comparable in both conditions, with a median of 67 nm (Figure 2C). These results imply that the extensive gain of chromatin loops in differentiating cells is not accompanied by notable changes in physical compaction of the euchromatic fraction of the genome.

CTCF Is Recruited to Anchors of Loops Induced upon Differentiation

The formation of cell-type-specific chromatin loops coincides with the context-dependent binding of CTCF and cohesin complex at loop anchors (Rao et al., 2014). To determine how changes in genomic occupancy of the two factors relate to loop dynamics, we mapped the binding sites of CTCF and cohesin subunit Rad21 by ChIP-seq. Consistent with recently published results (Beagan et al., 2017), we identified more CTCF peaks in ESCs than NSCs (61,560 and 44,848 CTCF peaks in ESCs and NSCs, respectively; Figure S3). We therefore hypothesized that (1) local gains of CTCF and Rad21, rather than a general increase in the number of CTCF-associated sites in NSCs, may underlie loop induction, (2) sites with elevated CTCF occupancy would be preferentially located at loop anchors, and (3) they would feature a CTCF-binding motif facing the interior of the loop (Rao et al., 2014; Vietri Rudan et al., 2015; de Wit et al., 2015).

We found NSC-specific loops to be associated with elevated occupancy of both CTCF and Rad21 at anchor sites (Figures 3A and 3B). We compared the distribution of increased (Benjamini-Hochberg adjusted $p < 0.1$; $FC > 1$; *DESeq2* method) CTCF peaks within domains demarcated by anchors of induced loops. We further stratified these by orientation of the CTCF DNA motif into forward and reverse groups. Peaks where CTCF signal increased in NSCs were located primarily at the edges of induced loop domains, facing the interior (Figure 3C). Thus, formation of chromatin loops is associated with increased CTCF and Rad21 binding at loop anchors. Peaks with enhanced CTCF signal are located preferentially at the edges of the induced loop domains, and CTCF motifs at these locations are oriented inward with respect to the interior of the loop.

Contact domain boundaries are primarily defined by CTCF binding sites and often coincide with loop anchors (Rao et al., 2014). We thus reasoned that increases in looping and CTCF binding would be related to the strength of domain boundaries. We considered 10-kb bins at contact domain boundaries that contained a CTCF peak (CTCF^{+} bins) and computed a measure of contact insulation (i.e., the ability to block chromosomal interactions (Sofueva et al., 2013) from our *in situ* Hi-C data (Figure 4A). CTCF^{+} bins at boundaries overlapping a loop anchor displayed significantly higher contact insulation than those not coincident with an anchor (Figure 4B; $p < 2.2 \times 10^{-16}$; two-sided t test). In line with this, we found that anchors of NSC-specific loops displayed increased contact insulation (Figure 4C; two-sided t test; $p < 2.2 \times 10^{-16}$), in contrast to the anchors of loops reduced in NSCs, where insulation was diminished (two-sided t test; $p = 1 \times 10^{-6}$). We conclude from these results that

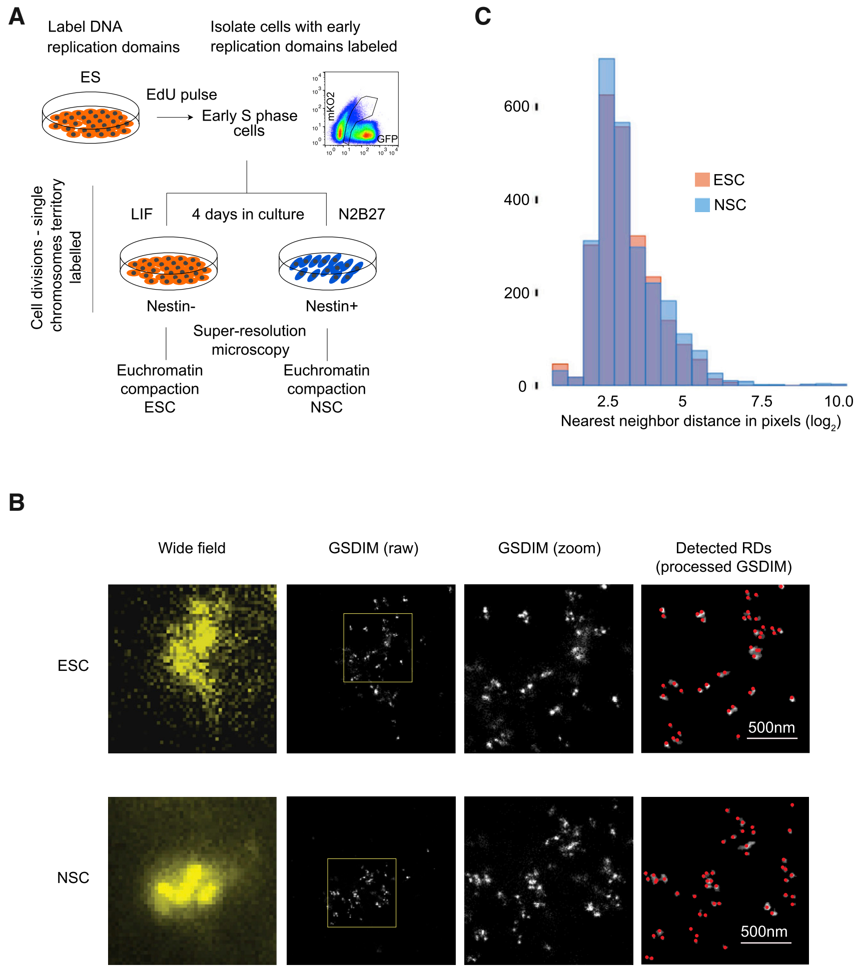

Figure 2. Compactness of Euchromatin Remains Unchanged upon Differentiation

(A) Experimental approach.

(B) SRI identification of RD in ESCs and Nestin$^+$ NSCs. Cells were labeled with anti-Nestin antibody prior to SRI, and Nestin$^-$ and Nestin$^+$ fractions were analyzed in ESC and post-neural induction cultures, respectively (Nestin signal not shown). RDs imaged by conventional microscopy (first panel column), GSDIM (pixel size 10 nm; second and third panel columns), and RD detection (fourth panel column) by automated image analysis.

(C) Nearest neighbor distance (NND) distributions in ESCs (red) and NSCs (blue) (sample sizes: $n_{ES} = 24$, $n_{NS} = 19$; RDs: $n_{ESC} = 2{,}410$, $n_{NSC} = 2{,}576$; pixel size = 10 nm).

induction of loop formation coincides with reinforcement of contact domain boundaries.

These data suggest that early lineage specification is accompanied by enhanced spatial segregation between neighboring regions of the genome. To test this idea, we extended our analysis to compare the strength of contact domain boundaries in differentiation. We considered boundaries identified via *in situ* Hi-C, which were frequently shared between ESCs and NSCs (60% common; Figures S4A and S4B). Cell-type-specific boundaries were weaker than those that were shared (Figures S4A and S4C) (Dixon et al., 2012). This result is consistent with the finding that both lost and gained boundaries were more frequently devoid of CTCF (Figure S4D). We found that boundaries common to both cell types were significantly weaker in ESCs (Figures 4D, S4C, and S4E; two-sided t test; $p < 2.2 \times 10^{-16}$) and that the increase in contact domain insulation upon differentiation was reflected by preferential recruitment of CTCF and Rad21 (Figure 4E).

Thus, early lineage specification is accompanied by enhanced spatial segregation between neighboring regions of the genome, and this correlates with a preferential gain of CTCF binding at contact domain boundaries. Accordingly, de-differentiating NSCs into induced pluripotent stem cells (iPSCs) by overexpression of exogenous Oct4, Nanog, Klf4, and Myc erased NSC-specific loops (Figure 4F). Furthermore, the loss of loop structures upon reprogramming resulted in significantly weaker contact domain boundaries in pluripotent cell cultures (Figure 4G, two-sided t test, $p < 2.2 \times 10^{-16}$). This is in keeping with previous observations that reversion to pluripotency affects the depletion of structural loops specific to the cell type of origin (Beagan et al., 2016; Krijger et al., 2016). Taken together, these observations establish weak contact domain boundaries as a hallmark of the pluripotent stem cell chromatin architecture.

Progressive Establishment of Chromatin Topology in Lineage Progression

We next asked whether the propensity to form loops and strong contact domain boundaries increases gradually or abruptly upon restriction of developmental potential. In the mouse, the transition from naive to primed pluripotency (Nichols and Smith, 2009) occurs concomitantly with uterine implantation of the embryo and is among the earliest cell fate decisions in mammalian development (Boroviak et al., 2014; Nichols and Smith, 2012). To emulate this transition *in vitro*, we obtained uniform populations of ESCs cultured in the presence of inhibitors of the MEK/ERK and glycogen synthase kinase 3 pathways (2i) plus LIF (Wray et al., 2011; Ying et al., 2008). From these, we derived primed pluripotent cell cultures (post-implantation epiblast stem cells [EpiSCs]) (Brons et al., 2007; Tesar et al., 2007) via exchange of 2i/LIF with FGF and activin followed by extended passaging (Figure 5A and STAR Methods). We generated TCC libraries from both cell types and found that 26% of chromatin loops displayed a significant change in strength (FDR = 0.1; FC > 1.5; Figure 5B). We observed that loops were 2.7 times more frequently gained than lost upon differentiation priming (840 versus 306; $n_{total} = 4{,}463$; Figure 5C), suggesting a gradual setup of chromatin structure accompanying exit from the naive state.

To evaluate this further, we sought to determine if chromatin loops induced in NSCs are similarly enhanced in EpiSCs. We considered loops for which we detected a significant increase in strength in NSC relative to ground-state ESC cultures

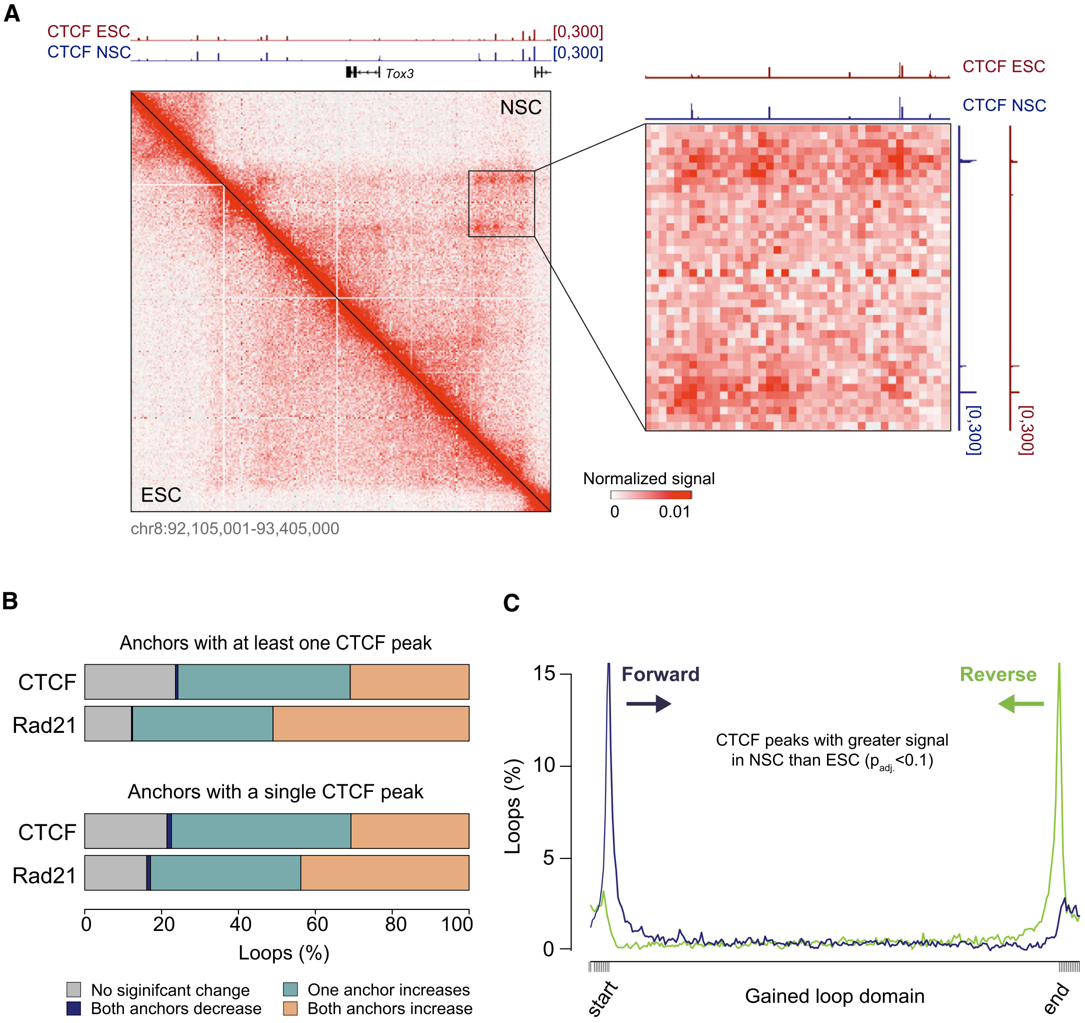

Figure 3. Loop Formation Is Associated with Gains in CTCF and Cohesin Binding

(A) Example of concomitant loop gain (*in situ* Hi-C) and increased CTCF ChIP-seq signal.

(B) Anchors of induced loops primarily overlap CTCF peaks that gain CTCF and Rad21 signal upon neural induction of ESCs. The union of CTCF peaks identified in ESCs and NSCs (P_{CTCF}) was considered. ChIP-seq reads were counted inside each P_{CTCF} interval, and differences were assessed with the *DESeq2* method. P_{CTCF} with $p_{adj.} < 0.1$, for which NSC/ESC > 1 were also considered gained. Top: loops for which both anchors overlapped at least one CTCF peak. Bottom: loops with a single CTCF peak at each anchor (n = 479, 20% of loops, consistent with Rao et al. [2014]).

(C) Loop induction correlates with a gain of CTCF peaks located primarily at loop anchors and facing the interior of the loop. Increased sites were those where the normalized ChIP-seq ratio of NSC/ESC was > 1 and $p_{adj.} < 0.1$ (*DESeq2* method). CTCF peaks were further stratified based on the orientation of the CTCF motif (forward and reverse groups). Each domain, defined by the anchors of an induced loop, was divided into 250 intervals (x axis; ten intervals were appended to the starts and ends of the loop domains), and the overlap with CTCF peaks was assessed therein. The percentage of domains intersecting a CTCF peak group is shown along the y axis.

maintained in 2i/LIF (Figure 5D; TCC data). Loop strength in EpiSCs fell between that of ESCs and NSCs, and this was the case for all stratifications by genomic distance (Figure 5E). To test whether an analogous stepwise progression also characterizes the gain of contact domain insulation, we considered induced loops and compared composite interaction profiles around anchors at those regions in naive and primed pluripotent cells and in NSCs. Indeed, we found insulation of chromatin contacts to be established gradually, concurrent with the loss of naive pluripotency (Figure 5F).

In summary, differentiation priming of pluripotent cells is reflected by a gain of chromatin loops and boundaries. Yet, the strength of these architectural features intensifies during specialization to a developmentally restricted multipotent stem cell type. Thus, chromatin topology is established progressively upon lineage commitment and cell-type transitions.

Gene Regulation and the Induction of Chromatin Loops

The interplay between structural loop formation and developmental control of gene expression is not well understood. Loops can bring together active promoters and enhancers (de Laat and Duboule, 2013; Rao et al., 2014). We considered our high-resolution *in situ* Hi-C maps in ESCs and NSCs to investigate how loop induction relates to gene expression control. We found that gained

OPEN
ACCESS
CellPress

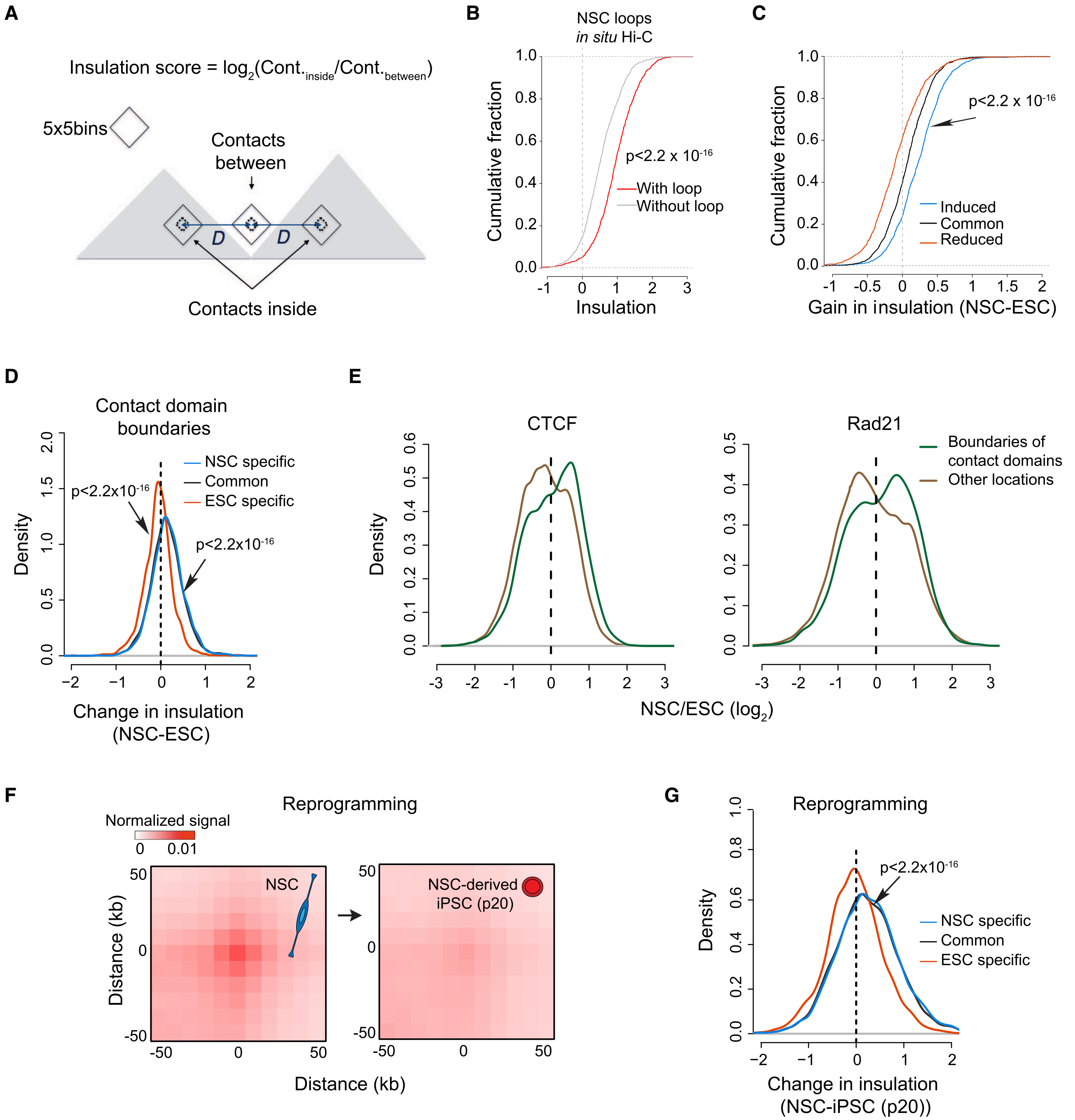

Figure 4. Pluripotent Stem Cell Chromatin Features Weak Chromatin Domain Boundaries

(A) Schema of the definition of the insulation score at a boundary between two domains (gray) as the $\log_2$ of the ratio of "inside" to "between" interactions. The score is positive for strong insulators and negative for weak insulators.

(B) Insulatory strength of CTCF sites at contact domain boundaries is correlated with loop formation. Bins overlapping a CTCF peak and at domain boundaries were stratified based on whether they overlapped with a loop anchor (with/without loop; p values: two-sided t test, NSCs, *in situ* Hi-C data).

(C) Difference of insulation scores (NSC minus ESC) at anchors of reduced, common, and induced loops ($p < 2.2 \times 10^{-16}$, two-sided t test; induced versus reduced loops, *in situ* Hi-C data).

(D) Boundaries of contact domains display overall lower insulation score in ESCs relative to differentiated cells.

(E) CTCF and Rad21 binding more frequently increases at boundaries of contact domains than at other genomic locations ($p < 2.2 \times 10^{-16}$, two-sided t test), which preferentially lose CTCF and Rad21 signals, consistent with the detection of greater numbers of peaks in ESCs.

(F) Reprogramming-induced depletion of loops; average of the Hi-C profiles (data from Krijger et al., 2016) at induced loops (*in situ* Hi-C data, n = 2,454) in NSCs and reprogrammed derivatives.

(G) Insulation scores at contact domain boundaries are diminished upon reversion of NSCs to iPSCs (two-sided t test).

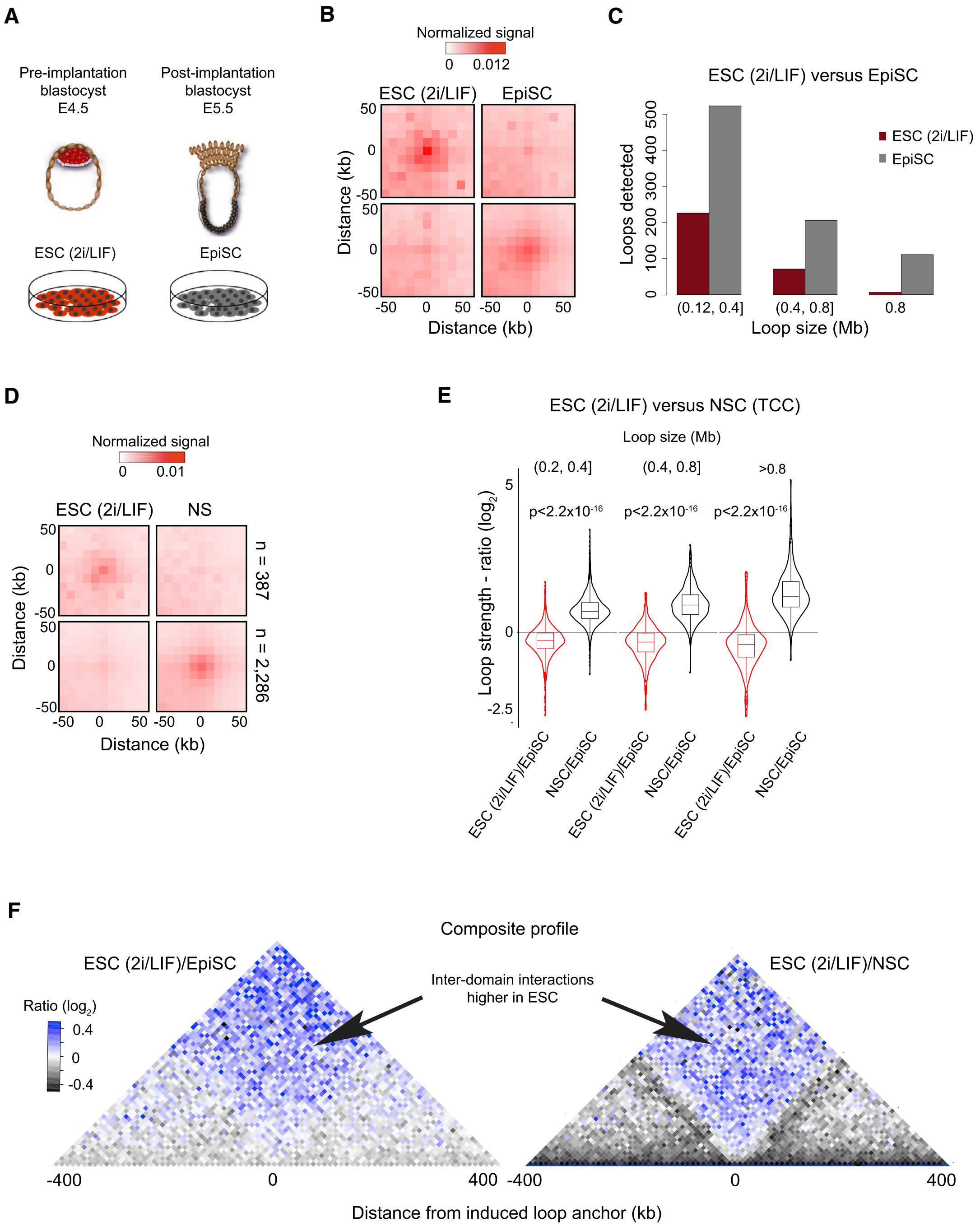

Figure 5. Chromatin Topology Is Established Progressively during Differentiation

(A) Experimental design: *in vitro* conditions to obtain uniform cultures of ground-state pluripotent cells (ESCs maintained in 2i/LIF) and primed pluripotent stem cells (post-implantation epiblast stem cells [EpiSCs]).

(B) Composite profile of TCC signal at loops identified as stronger in ESCs (top) or EpiSCs (bottom).

(C) Length distribution of loops specific to ESCs and EpiSCs.

(D) Composite profile of loops displaying a significant alteration of TCC signal between ESCs (2i/LIF) and NSCs. Loops identified in either or both conditions were considered (TCC data).

(E) Loops are gained in a stepwise manner following loss of naive pluripotency. Loops identified as induced in NSCs relative to ESCs (2i/LIF) were considered (TCC data). Induced loops were grouped into three classes according to genomic span. For each class, ratios of the loop signal between ESCs or NSCs to the signal in EpiSCs are displayed. Loop strength in EpiSCs is between that of ESCs and NSCs (two-sided t test).

(F) Interactions across anchors of NSC-specific loops are gradually lost. The two panels display the ratios between composite profiles of the TCC signal around anchors of induced loops (ESCs [2i/LIF] versus NSCs; TCC data) at 10-kb resolution. Left: ratio of ESC (2i/LIF) to NSC TCC signal; right plot: ratio of ESCs to EpiSCs.

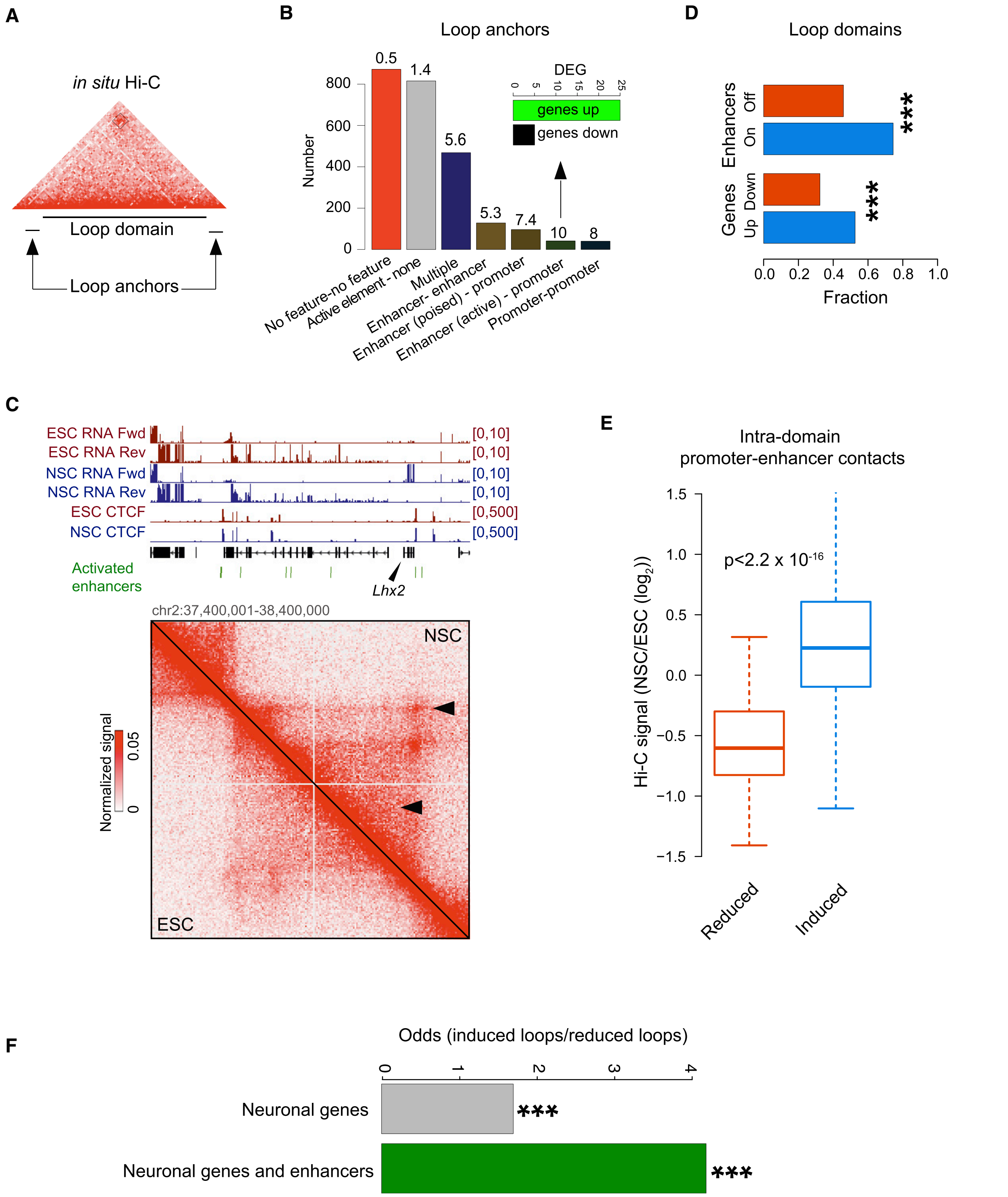

Figure 6. Loop Dynamics and the Regulation of Gene Expression

(A) Loop domains are genomic intervals defined by the end of the left anchor (+10 kb) and the start of the right anchor (−10 kb).

(B) Induced loops (*in situ* Hi-C; n = 2,454) preferentially connect active regulatory elements. Enrichment relative to random pairs of loci separated by a similar genomic distance is indicated above each bar. Inset: the number of up- and downregulated genes (*DESeq* method; FC > 1.5; adjusted $p < 0.1$) among loci with promoters forming a loop with enhancers in NSCs only.

(C) Example of an upregulated locus (*Lhx2*) inside an induced loop domain.

(legend continued on next page)

loops frequently involved active regulatory elements (Figures 6A, 6B, and S5A). Genes with promoters overlapping the anchors of NSC-specific loops were functionally related to brain development (Fisher's exact test, Benjamini-Hochberg [BH] corrected $p < 0.01$ and fold enrichment [odds ratio] ≥ 1.5; Figure S5B). Compared to transcriptionally downregulated genes, upregulated loci (>1.5-fold; FDR = 0.1) were overrepresented at anchors of induced promoter-enhancer loops ($p = 3.2 \times 10^{-4}$; binomial test; Figures 6B and S5A). However, induction of gene expression was directly linked with only a minority of gained loops (552 out of 2,454, 22%). Notably, many loops that were formed upon neural induction did not involve promoter regions (Figure 6C).

According to the insulated neighborhoods model, regulation of genes critical to the establishment of cell identity occurs frequently in chromatin domains defined by cohesin-mediated interactions (Dowen et al., 2014). Given that cohesin binding is primarily detected at CTCF binding sites (Parelho et al., 2008; Rubio et al., 2008; Stedman et al., 2008; Wendt et al., 2008), we hypothesized that domains flanked by differentiation-induced loops (Figure 6A) may encompass loci implicated in neuroectoderm fate. Indeed, 74% of induced loop domains contained enhancers activated in NSCs, as determined by gains in H3K27ac modification. Upregulation of genes within those domains was twice as frequent as downregulation (Figure 6D; 1,250 versus 852, >1.5-fold; FDR = 0.1; *DESeq* method). For example, the neuronal gene *Lhx2* is expressed *de novo* in NSCs and located inside an induced loop domain (Figure 6C). We consistently observed enrichment of Gene Ontology functional terms related to neural development within genomic intervals delineated by induced loops (Fisher's exact test; BH corrected $p < 0.01$ and fold increase ≥ 1.5; Figure S5C). These results suggest that chromatin loop formation is involved in the control of genes associated with cell identity.

Contact domains can be subdivided into ordinary and loop domains (Rao et al., 2014). Yet, it is unclear whether the presence of a loop is related to the strength of intra-domain interactions, including those between regulatory elements. We found that contacts in loop domains were on average stronger than in ordinary domains (Figure S5D). Moreover, promoter-enhancer interactions (PEIs) that connected elements located *inside* the induced loop domains increased upon differentiation, whereas PEIs linking elements in dismantled loops were significantly decreased (Figures 6C and 6E; $p < 2.2 \times 10^{-16}$; two-sided t test). Hence, the formation of a chromatin loop reflects not only increased separation of the genomic interval enclosed by its anchors but is also coupled to a gain of contacts between enhancers and promoters. The regulatory element pairs either overlap the two loop anchors or are located inside the loop domain. These findings echo those reported from experiments in B cells, where activation was linked with an increase in the number of loops detected and a gain of intra-domain promoter-enhancer contacts (Kieffer-Kwon et al., 2017).

By visual inspection of *in situ* Hi-C interactome profiles with respect to transcriptional activity, we saw that the relationship between loop induction and gene expression was frequently more complex than a direct positive correlation. Notably, we found many examples of loci within an induced loop despite being transcriptionally inactive in NSCs. *Opcml* and *Kcnc2* (Figure S6) exemplify this finding. These genes are transcriptionally active in differentiated neural tissues: *Opcml* is widely expressed throughout the brain and in the retina, and *Kcnc2* is primarily expressed in the cortex (Su et al., 2004). Thus, our data are in line with the concept that loops are involved in architectural priming for future gene expression (de Laat and Duboule, 2013). Supporting this view, genes critical for adult brain function were significantly enriched among loci flanked by anchors of induced loops (Figure S5C). Moreover, induced loop domains contained pairs of genes and enhancers that are active in adult brain tissues more often than reduced loop domains. (Figure 6F).

We also considered the possibility that loops may have arisen at these loci due to transcriptional activation of surrounding genes located within the domain. However, expression levels of neighboring genes were frequently unaltered in ESCs and NSCs (Figure S6). In contrast, there was no enrichment of either developmental genes or pluripotency-related terms among genes spanning domains defined by loops that were lost or reduced in response to differentiation. In fact, domains encompassing pluripotency-associated loci (e.g., *Oct4* and *Prdm14*) were either depleted of long-range loops in ESCs (Figure S7) or enriched in loops that were short range and stable across conditions (e.g., *Nanog*). Thus, structural loops arising upon differentiation are preferentially associated with genes that impart cell-type specification.

In summary, differentiation-induced formation of long-range structural loops is affected through local recruitment of CTCF at loop anchors. Such changes in genome structure act to spatially segregate chromatin interaction domains containing genes and regulatory elements implicated in lineage choice and embryonic development.

DISCUSSION

Here, we investigate the topological features of genome architecture in pluripotent cells and specialized progeny. Using a combination of chromatin conformation capture and SRI, we show that structural changes manifest progressively in cell differentiation to establish long-range loops and chromatin boundaries. These domains often encompass developmentally related genes upregulated in response to lineage induction and are consequently dismantled in cells that have undergone reversion to pluripotency through transgene-mediated reprogramming. We further identify CTCF and Rad21 as mediators of architectural remodeling and spatial segregation of chromatin interaction domains. These insights support and extend related findings in diverse model systems and clarify the role of chromatin loops in the developmental control of gene expression.

(D) Induced loop domains are formed around activated enhancers and upregulated genes. The x axis plots the fraction of induced loop domains overlapping induced and repressed enhancers (top) and transcriptionally up- and downregulated genes (bottom).

(E) Loop changes correlate with the dynamics of intra-loop-domain promoter-enhancer contacts measured by *in situ* Hi-C (two-sided t test).

(F) Genes and enhancers active in adult neuronal tissues are found more frequently inside induced than reduced loop domains (Fisher's exact test).

Table 1. Findings from This Work and Related Studies

Feature	This study	Bonev et al. (2017)	Stadhouders et al. (2018)
In vitro systems	Embryonic stem cells (ESCs) (conventional and ground-state cultures), post-implantation epiblast stem cells (EpiSCs), induced pluripotent stem cells (iPSCs), neural stem cells (NSCs).	ESCs (conventional), neural progenitor cells (NPCs) and post-mitotic cortical neurons (CNs). NPCs and CNs were derived *in vitro* or isolated from neocortex.	Reprogramming of B cells to iPSCs.
CTCF-anchored loops	Stepwise genome-wide induction of long-range loops upon exit from naive pluripotency.	Genome-wide gain of contacts between domain boundaries and convergent CTCF binding sites.	Dissolution of B cell-specific loops after reprogramming. iPSC-specific loops identified as long range.
Domain type	Contact domains; topologically associated domains (TADs); replication domains	TADs	TADs
Domain boundaries in cell differentiation	Progressive strengthening following loss of naive pluripotency and differentiation to EpiSCs and more specialized NSCs.	Strengthening upon differentiation of ESCs to NPCs. Pronounced strengthening of domain boundaries was not observed in terminally differentiated cells.	N/A
Domain boundaries in reprogramming	Restores weak domain boundaries.	N/A	Gain of domain boundaries is more frequent than loss after reprogramming.
Chromatin compaction	Not coupled with genome-wide gain of loops.	N/A	N/A
Profiles of CTCF and cohesins	More peaks in ESCs than NSCs. Neural induction results in quantitative gain of CTCF, and cohesin binding at loop anchors and domain boundaries. Binding of the two factors is diminished at other genomic loci.	Presence/absence of CTCF peaks does not account for genome-wide gain of loops and domain boundary strength.	CTCF binding correlates with insulatory strength of TAD boundaries. No correlation between gain or loss of TAD boundaries and CTCF recruitment.
Relationship between gene expression, chromatin domains, domain boundaries, and structural loops	Positive correlation between loop formation and gene expression. Few loops connect active promoters and enhancers. Induced loop domains span enhancers and developmentally regulated genes.	Positive correlation between loop formation and gene expression. Transcriptional activation frequently coincides with formation of boundaries but is insufficient to elicit boundary formation.	Positive correlation between loop formation and gene expression. No correlation between formation of TAD boundaries and transcriptional regulation. Changes in TAD structure precede gene activation.

Recent studies have revealed a lack of chromosome-scale A/B compartments in the early embryos of *Drosophila* (Hug et al., 2017) and in the maternal genome of the mouse zygote (Flyamer et al., 2017). In the mouse, TADs and loops are detectable in zygotic maternal chromatin, in contrast to the features reported in fly (Hug et al., 2017). However, it has now been shown that TADs consolidate upon the transition between 2- and 8-cell stage mouse embryos (Du et al., 2017; Ke et al., 2017). We observed a lower prevalence of long-range structural loops, accompanied by decreased insulation, as a distinguishing feature of pluripotent chromatin. These observations are consistent with reports describing enhanced genome-wide clustering of active regulatory elements in ESCs (Novo et al., 2018; de Wit et al., 2013) and decreased intra-domain connectivity in iPSCs as compared to B cells (Table 1) (Stadhouders et al., 2018). Our SRI data indicate that the structural changes we and others have observed do not arise as a result of chromatin compaction. We argue for a central role of CTCF in establishing the unique nuclear topology of pluripotent stem cells.

The study we present here is one of three investigating alterations to chromatin topology related to changes in cell identity upon differentiation and reprogramming (Table 1). Bonev et al. (2017) described qualitative differences in CTCF binding between ESCs and NSCs, correlating CTCF occupancy with changes in chromatin loops resolved in each cell type. Contacts were observed to increase in NSCs between TAD boundaries and, more generally, between convergent CTCF binding sites (Bonev et al., 2017). Domain boundaries shared between ESCs and NSCs were determined to be weaker in the pluripotent state. Our findings corroborate those of Bonev et al. (2017) (Table 1) but are derived through a different strategy. We identify genome-wide chromatin loops induced or diminished in response to ESC differentiation. We then map CTCF and Rad21 binding activity and relate those differences in chromatin architecture to cohesins and gene expression. We further investigate loops arising around developmentally regulated genes and active enhancers. This approach enabled us to infer dynamic changes in loop formation and reinforcement and to gauge the influence of those changes on transcriptional regulation of lineage induction and, conversely, reversion to pluripotency.

We show that chromatin loops and strong domain boundaries are formed progressively from the exit from naive pluripotency through commitment to the neural lineage. Interestingly, terminal differentiation of neural progenitor cells does not appear to further promote the frequency of contacts between convergent CTCF sites (Bonev et al., 2017), favoring a model in which the loss of pluripotency marks a critical transition required to fully establish loops and chromatin boundaries. In light of the data collected to date, the cause and consequence of these observations remain unresolved. Using Hi-C, Graff and colleagues recently resolved the trajectory of the alterations to chromatin organization during reprogramming at fine temporal resolution (Stadhouders et al., 2018). That analysis revealed that structural reorganization of chromatin frequently precedes transcriptional changes. This finding suggests an instructive role for genome topology in cell fate transitions. Accordingly, we speculate that increases in looping and boundary strength may be a precursor to the dissolution of the pluripotency regulatory network. In the future, it will be important to elucidate how the presence of strong contact domain (including TAD) boundaries and long-range chromatin loops relates to cell identity. Addressing these and related aspects of differentiation and fate choice will be central to understanding the role of higher-order genome architecture in the regulation of mammalian development.

STAR★METHODS

Detailed methods are provided in the online version of this paper and include the following:

- KEY RESOURCES TABLE
- CONTACT FOR REAGENT AND RESOURCE SHARING
- EXPERIMENTAL MODEL AND SUBJECT DETAILS
  - Cell Culture Conditions
- METHOD DETAILS
  - Analysis by Flow Cytometry
  - ChIP-Seq
  - Tethered Chromatin Conformation
  - In-Situ Hi-C
  - RNA-Seq
  - Super-Resolution Imaging
- QUANTIFICATION AND STATISTICAL ANALYSIS
  - High-Throughput Sequencing Data Analysis
- DATA AND SOFTWARE AVAILABILITY

SUPPLEMENTAL INFORMATION

Supplemental Information includes seven figures and one table and can be found with this article online at https://doi.org/10.1016/j.cels.2018.09.003.

ACKNOWLEDGMENTS

We thank Hüseyin Besir of the EMBL Protein Expression and Purification Facility for custom growth factor production; Vladimir Benes and Bettina Haase of the EMBL Genomics Core Facility for library preparation and sequencing; and Andrew Riddell and Alexis Gonzalez of the EMBL Flow Cytometry Core Facility. FUCCI reporter cells were kindly provided by Matthias Lutolf. We thank Steven Pollard for NSC protocols; Florian Hahne for help with data visualization; and Jens Kalchschmidt for a critical reading of the manuscript. The J.E. lab was supported by EMBL and the US National Institutes of Health (NIH) through the 4D Nucleome (4DN) common fund (U01 EB021223, U01 DA047728). The P.B. lab was supported by EMBL and the UK Biotechnology and Biological Sciences Research Council (BBSRC) (BB/M004023/1, BB/G015678/1), with core funding from the Wellcome Trust and the UK Medical Research Council (MRC) (203151/Z/16/Z, 097922/Z/11/Z). The W.H. lab was supported by EMBL and the European Commission Seventh Framework Programme (FP7) through the collaborative research project RADIANT (305626). A.P. was supported by an EMBL Interdisciplinary Postdoc (EIPOD) fellowship under Marie Curie Actions COFUND. F.A.K., M.O., and W.X. were supported by the EMBL International PhD Programme (EIPP).

AUTHOR CONTRIBUTIONS

A.P. and W.H. designed the study with input from P.B. and L.M.S. A.P. and J.S. performed all experiments except for super-resolution imaging, which was carried out by W.X., N.D., and J.E. P.B. and R.C. contributed reagents, experimental setup, and design. A.P. and B.K. analyzed the data in consultation with W.H. and P.B. F.A.K. and M.O. contributed to initial data processing. W.H., P.B., and L.M.S. supervised the study. A.P., P.B., and W.H. wrote the manuscript.

DECLARATION OF INTERESTS

The authors declare no competing interests.

Received: July 20, 2017
Revised: March 6, 2018
Accepted: September 24, 2018
Published: November 7, 2018

REFERENCES

Anders, S., and Huber, W. (2010). Differential expression analysis for sequence count data. Genome Biol. *11*, R106.

Anders, S., Pyl, P.T., and Huber, W. (2015). HTSeq–a python framework to work with high-throughput sequencing data. Bioinformatics *31*, 166–169.

Apostolou, E., Ferrari, F., Walsh, R.M., Bar-Nur, O., Stadtfeld, M., Cheloufi, S., Stuart, H.T., Polo, J.M., Ohsumi, T.K., Borowsky, M.L., et al. (2013). Genome-wide chromatin interactions of the Nanog locus in pluripotency, differentiation, and reprogramming. Cell Stem Cell *12*, 699–712.

Banterle, N., Bui, K.H., Lemke, E.A., and Beck, M. (2013). Fourier ring correlation as a resolution criterion for super-resolution microscopy. J. Struct. Biol. *183*, 363–367.

Beagan, J.A., Gilgenast, T.G., Kim, J., Plona, Z., Norton, H.K., Hu, G., Hsu, S.C., Shields, E.J., Lyu, X., Apostolou, E., et al. (2016). Local genome topology can exhibit an incompletely rewired 3D-folding state during somatic cell reprogramming. Cell Stem Cell *18*, 611–624.

Beagan, J.A., Duong, M.T., Titus, K.R., Zhou, L., Cao, Z., Ma, J., Lachanski, C.V., Gillis, D.R., and Phillips-Cremins, J.E. (2017). YY1 and CTCF orchestrate a 3D chromatin looping switch during early neural lineage commitment. Genome Res. *27*, 1139–1152.

Bickmore, W.A., and van Steensel, B. (2013). Genome architecture: domain organization of interphase chromosomes. Cell *152*, 1270–1284.

Bonev, B., Mendelson Cohen, N., Szabo, Q., Fritsch, L., Papadopoulos, G.L., Lubling, Y., Xu, X., Lv, X., Hugnot, J.P., Tanay, A., et al. (2017). Multiscale 3D genome rewiring during mouse neural development. Cell *171*, 557–572.e24.

Boroviak, T., Loos, R., Bertone, P., Smith, A., and Nichols, J. (2014). The ability of inner-cell-mass cells to self-renew as embryonic stem cells is acquired following epiblast specification. Nat. Cell Biol. *16*, 516–528.

Brons, I.G., Smithers, L.E., Trotter, M.W., Rugg-Gunn, P., Sun, B., Chuva de Sousa Lopes, S.M., Howlett, S.K., Clarkson, A., Ahrlund-Richter, L., Pedersen, R.A., et al. (2007). Derivation of pluripotent epiblast stem cells from mammalian embryos. Nature *448*, 191–195.

Conti, L., Pollard, S.M., Gorba, T., Reitano, E., Toselli, M., Biella, G., Sun, Y., Sanzone, S., Ying, Q.L., Cattaneo, E., et al. (2005). Niche-independent symmetrical self-renewal of a mammalian tissue stem cell. PLoS Biol. *3*, e283.

Deng, W., Rupon, J.W., Krivega, I., Breda, L., Motta, I., Jahn, K.S., Reik, A., Gregory, P.D., Rivella, S., Dean, A., et al. (2014). Reactivation of developmentally silenced globin genes by forced chromatin looping. Cell *158*, 849–860.

Denholtz, M., Bonora, G., Chronis, C., Splinter, E., de Laat, W., Ernst, J., Pellegrini, M., and Plath, K. (2013). Long-range chromatin contacts in embryonic stem cells reveal a role for pluripotency factors and Polycomb proteins in genome organization. Cell Stem Cell *13*, 602–616.

Dixon, J.R., Selvaraj, S., Yue, F., Kim, A., Li, Y., Shen, Y., Hu, M., Liu, J.S., and Ren, B. (2012). Topological domains in mammalian genomes identified by analysis of chromatin interactions. Nature *485*, 376–380.

Dixon, J.R., Jung, I., Selvaraj, S., Shen, Y., Antosiewicz-bourget, J.E., Lee, A.Y., Ye, Z., Kim, A., Rajagopal, N., Xie, W., et al. (2015). Chromatin architecture reorganization during stem cell differentiation. Nature *518*, 331–336.

Dowen, J.M., Fan, Z.P., Hnisz, D., Ren, G., Abraham, B.J., Zhang, L.N., Weintraub, A.S., Schujiers, J., Lee, T.I., Zhao, K., et al. (2014). Control of cell identity genes occurs in insulated neighborhoods in mammalian chromosomes. Cell *159*, 374–387.

Du, Z., Zheng, H., Huang, B., Ma, R., Wu, J., Zhang, X., He, J., Xiang, Y., Wang, Q., Li, Y., et al. (2017). Allelic reprogramming of 3D chromatin architecture during early mammalian development. Nature *547*, 232–235.

Durand, N.C., Shamim, M.S., Machol, I., Rao, S.S.P., Huntley, M.H., Lander, E.S., and Aiden, E.L. (2016a). Juicer provides a one-click system for analyzing loop-resolution Hi-C experiments. Cell Syst. *3*, 95–98.

Durand, N.C., Robinson, J.T., Shamim, M.S., Machol, I., Mesirov, J.P., Lander, E.S., and Aiden, E.L. (2016b). Juicebox provides a visualization system for Hi-C contact maps with unlimited zoom. Cell Syst. *3*, 99–101.

Efroni, S., Duttagupta, R., Cheng, J., Dehghani, H., Hoeppner, D.J., Dash, C., Bazett-Jones, D.P., Le Grice, S., McKay, R.D.G., Buetow, K.H., et al. (2008). Global transcription in pluripotent embryonic stem cells. Cell Stem Cell *2*, 437–447.

Ernst, J., and Kellis, M. (2012). ChromHMM: automating chromatin-state discovery and characterization. Nat. Methods *9*, 215–216.

Flavahan, W.A., Drier, Y., Liau, B.B., Gillespie, S.M., Venteicher, A.S., Stemmer-Rachamimov, A.O., Suvà, M.L., and Bernstein, B.E. (2016). Insulator dysfunction and oncogene activation in IDH mutant gliomas. Nature *529*, 110–114.

Flyamer, I.M., Gassler, J., Imakaev, M., Brandão, H.B., Ulianov, S.V., Abdennur, N., Razin, S.V., Mirny, L.A., and Tachibana-Konwalski, K. (2017). Single-nucleus Hi-C reveals unique chromatin reorganization at oocyte-to-zygote transition. Nature *544*, 110–114.

Grant, C.E., Bailey, T.L., and Noble, W.S. (2011). FIMO: scanning for occurrences of a given motif. Bioinformatics *27*, 1017–1018.

Huang, da W., Sherman, B.T., and Lempicki, R.A. (2009). Systematic and integrative analysis of large gene lists using David bioinformatics resources. Nat. Protoc. *4*, 44–57.

Huber, W., von Heydebreck, A., Sueltmann, H., Poustka, A., and Vingron, M. (2003). Parameter estimation for the calibration and variance stabilization of microarray data. Stat. Appl. Genet. Mol. Biol. *2*, Article3.

Hug, C.B., Grimaldi, A.G., Kruse, K., and Vaquerizas, J.M. (2017). Chromatin architecture emerges during zygotic genome activation independent of transcription. Cell *169*, 216–228.e19.

Imakaev, M., Fudenberg, G., Mccord, R.P., Naumova, N., Goloborodko, A., Lajoie, B.R., Dekker, J., and Mirny, L.A. (2012). Iterative correction of Hi-C data reveals hallmarks of chromosome organization. Nat. Methods *9*, 999–1003.

Irizarry, R.A., Hobbs, B., Collin, F., Beazer-Barclay, Y.D., Antonellis, K.J., Scherf, U., and Speed, T.P. (2003). Exploration, normalization, and summaries of high density oligonucleotide array probe level data. Biostatistics *4*, 249–264.

Kalhor, R., Tjong, H., Jayathilaka, N., Alber, F., and Chen, L. (2011). Genome architectures revealed by tethered chromosome conformation capture and population-based modeling. Nat. Biotechnol. *30*, 90–98.

Ke, Y., Xu, Y., Chen, X., Feng, S., Liu, Z., Sun, Y., Yao, X., Li, F., Zhu, W., Gao, L., et al. (2017). 3D chromatin structures of mature gametes and structural reprogramming during mammalian embryogenesis. Cell *170*, 367–381.e20.

Kieffer-Kwon, K.R., Tang, Z., Mathe, E., Qian, J., Sung, M.H., Li, G., Resch, W., Baek, S., Pruett, N., Grøntved, L., et al. (2013). Interactome maps of mouse gene regulatory domains reveal basic principles of transcriptional regulation. Cell *155*, 1507–1520.

Kieffer-Kwon, K.R., Nimura, K., Rao, S.S.P., Xu, J., Jung, S., Pekowska, A., Dose, M., Stevens, E., Mathe, E., Dong, P., et al. (2017). Myc regulates chromatin decompaction and nuclear architecture during B cell activation. Mol. Cell *67*, 566–578.e10.

Kim, D., Pertea, G., Trapnell, C., Pimentel, H., Kelley, R., and Salzberg, S.L. (2013). TopHat2: accurate alignment of transcriptomes in the presence of insertions, deletions and gene fusions. Genome Biol. *14*, R36.

Krijger, P.H.L., Di Stefano, B., de Wit, E., Limone, F., van Oevelen, C., de Laat, W., and Graf, T. (2016). Cell-of-origin-specific 3D genome structure acquired during somatic cell reprogramming. Cell Stem Cell *18*, 597–610.

de Laat, W., and Duboule, D. (2013). Topology of mammalian developmental enhancers and their regulatory landscapes. Nature *502*, 499–506.

Langmead, B., and Salzberg, S.L. (2012). Fast gapped-read alignment with Bowtie 2. Nat. Methods *9*, 357–359.

Lieberman-Aiden, E., van Berkum, N.L., Williams, L., Imakaev, M., Ragoczy, T., Telling, A., Amit, I., Lajoie, B.R., Sabo, P.J., Dorschner, M.O., et al. (2009). Comprehensive mapping of long-range interactions reveals folding principles of the human genome. Science *326*, 289–293.

Love, M.I., Huber, W., and Anders, S. (2014). Moderated estimation of fold change and dispersion for RNA-seq data with DESeq2. Genome Biol. *15*, 1–34.

Lupiáñez, D.G., Kraft, K., Heinrich, V., Krawitz, P., Brancati, F., Klopocki, E., Horn, D., Kayserili, H., Opitz, J.M., Laxova, R., et al. (2015). Disruptions of topological chromatin domains cause pathogenic rewiring of gene-enhancer interactions. Cell *161*, 1012–1025.

Marks, H., Kalkan, T., Menafra, R., Denissov, S., Jones, K., Hofemeister, H., Nichols, J., Kranz, A., Stewart, A.F., Smith, A., et al. (2012). The transcriptional and epigenomic foundations of ground state pluripotency. Cell *149*, 590–604.

Mikkelsen, T.S., Ku, M., Jaffe, D.B., Issac, B., Lieberman, E., Giannoukos, G., Alvarez, P., Brockman, W., Kim, T.K., Koche, R.P., et al. (2007). Genome-wide maps of chromatin state in pluripotent and lineage-committed cells. Nature *448*, 553–560.

Nichols, J., and Smith, A. (2009). Naive and primed pluripotent states. Cell Stem Cell *4*, 487–492.

Nichols, J., and Smith, A. (2012). Pluripotency in the embryo and in culture. Cold Spring Harb. Perspect. Biol. *4*, a008128.

Nora, E.P., Lajoie, B.R., Schulz, E.G., Giorgetti, L., Okamoto, I., Servant, N., Piolot, T., van Berkum, N.L., Meisig, J., Sedat, J., et al. (2012). Spatial partitioning of the regulatory landscape of the X-inactivation centre. Nature *485*, 381–385.

Nora, E.P., Goloborodko, A., Valton, A.L., Gibcus, J.H., Uebersohn, A., Abdennur, N., Dekker, J., Mirny, L.A., and Bruneau, B.G. (2017). Targeted degradation of CTCF decouples local insulation of chromosome domains from genomic compartmentalization. Cell *169*, 930–944.e22.

Novo, C.L., Javierre, B.M., Cairns, J., Segonds-Pichon, A., Wingett, S.W., Freire-Pritchett, P., Furlan-Magaril, M., Schoenfelder, S., Fraser, P., and Rugg-Gunn, P.J. (2018). Long-range enhancer interactions are prevalent in mouse embryonic stem cells and are reorganized upon pluripotent state transition. Cell Rep. *22*, 2615–2627.

Parelho, V., Hadjur, S., Spivakov, M., Leleu, M., Sauer, S., Gregson, H.C., Jarmuz, A., Canzonetta, C., Webster, Z., Nesterova, T., et al. (2008). Cohesins functionally associate with CTCF on mammalian chromosome arms. Cell *132*, 422–433.

Phillips-Cremins, J.E., Sauria, M.E., Sanyal, A., Gerasimova, T.I., Lajoie, B.R., Bell, J.S., Ong, C.T., Hookway, T.A., Guo, C., Sun, Y., et al. (2013). Architectural protein subclasses shape 3D organization of genomes during lineage commitment. Cell *153*, 1281–1295.

Pukelsheim, F., and Simeone, B. (2009). On the iterative proportional fitting procedure: structure of accumulation points and L1-error analysis. https://opus.bibliothek.uni-augsburg.de/opus4/frontdoor/deliver/index/docId/1229/file/mpreprint_09_005.pdf.

Rao, S.S.P., Huntley, M.H., Durand, N.C., Stamenova, E.K., Bochkov, I.D., Robinson, J.T., Sanborn, A.L., Machol, I., Omer, A.D., Lander, E.S., et al. (2014). A 3D map of the human genome at kilobase resolution reveals principles of chromatin looping. Cell *159*, 1665–1680.

Ricci, M.A., Manzo, C., García-Parajo, M.F., Lakadamyali, M., and Cosma, M.P. (2015). Chromatin fibers are formed by heterogeneous groups of nucleosomes in vivo. Cell *160*, 1145–1158.

Robinson, M.D., McCarthy, D.J., and Smyth, G.K. (2010). edgeR: a bioconductor package for differential expression analysis of digital gene expression data. Bioinformatics *26*, 139–140.

Roccio, M., Schmitter, D., Knobloch, M., Okawa, Y., Sage, D., and Lutolf, M.P. (2013). Predicting stem cell fate changes by differential cell cycle progression patterns. Development *140*, 459–470.

Rubio, E.D., Reiss, D.J., Welcsh, P.L., Disteche, C.M., Filippova, G.N., Baliga, N.S., Aebersold, R., Ranish, J.A., and Krumm, A. (2008). CTCF physically links cohesin to chromatin. Proc. Natl. Acad. Sci. U S A *105*, 8309–8314.

Sanyal, A., Lajoie, B.R., Jain, G., and Dekker, J. (2012). The long-range interaction landscape of gene promoters. Nature *489*, 109–113.

Sexton, T., Yaffe, E., Kenigsberg, E., Bantignies, F., Leblanc, B., Hoichman, M., Parrinello, H., Tanay, A., and Cavalli, G. (2012). Three-dimensional folding and functional organization principles of the Drosophila genome. Cell *148*, 458–472.

Shen, Y., Yue, F., McCleary, D.F., Ye, Z., Edsall, L., Kuan, S., Wagner, U., Dixon, J., Lee, L., Lobanenkov, V.V., et al. (2012). A map of the cis-regulatory sequences in the mouse genome. Nature *488*, 116–120.

Sofueva, S., Yaffe, E., Chan, W.C., Georgopoulou, D., Vietri Rudan, M., Mira-Bontenbal, H., Pollard, S.M., Schroth, G.P., Tanay, A., and Hadjur, S. (2013). Cohesin-mediated interactions organize chromosomal domain architecture. EMBO J. *32*, 3119–3129.

Spitz, F. (2016). Gene regulation at a distance: From remote enhancers to 3D regulatory ensembles. Semin. Cell Dev. Biol. *57*, 57–67.

Stadhouders, R., Vidal, E., Serra, F., Di Stefano, B., Le Dily, F., Quilez, J., Gomez, A., Collombet, S., Berenguer, C., Cuartero, Y., et al. (2018). Transcription factors orchestrate dynamic interplay between genome topology and gene regulation during cell reprogramming. Nat. Genet. *50*, 238–249.

Stedman, W., Kang, H., Lin, S., Kissil, J.L., Bartolomei, M.S., and Lieberman, P.M. (2008). Cohesins localize with CTCF at the KSHV latency control region and at cellular c-myc and H19/Igf2 insulators. EMBO J. *27*, 654–666.

Su, A.I., Wiltshire, T., Batalov, S., Lapp, H., Ching, K.A., Block, D., Zhang, J., Soden, R., Hayakawa, M., Kreiman, G., et al. (2004). A gene atlas of the mouse and human protein-encoding transcriptomes. Proc. Natl. Acad. Sci. U S A *101*, 6062–6067.

Symmons, O., Uslu, V.V., Tsujimura, T., Ruf, S., Nassari, S., Schwarzer, W., Ettwiller, L., and Spitz, F. (2014). Functional and topological characteristics of mammalian regulatory domains. Genome Res. *24*, 390–400.

Symmons, O., Pan, L., Remeseiro, S., Aktas, T., Klein, F., Huber, W., and Spitz, F. (2016). The shh topological domain facilitates the action of remote enhancers by reducing the effects of genomic distances. Dev. Cell *39*, 529–543.

Szymborska, A., de Marco, A., Daigle, N., Cordes, V.C., Briggs, J.A.G., and Ellenberg, J. (2013). Nuclear pore scaffold structure analyzed by super-resolution microscopy and particle averaging. Science *341*, 655–658.

Tesar, P.J., Chenoweth, J.G., Brook, F.A., Davies, T.J., Evans, E.P., Mack, D.L., Gardner, R.L., and Mckay, R.D.G. (2007). New cell lines from mouse epiblast share defining features with human embryonic stem cells. Nature *448*, 196–199.

Tsujimura, T., Klein, F.A., Langenfeld, K., Glaser, J., Huber, W., and Spitz, F. (2015). A discrete transition zone organizes the topological and regulatory autonomy of the adjacent tfap2c and bmp7 genes. PLoS Genet. *11*, e1004897.

Vietri Rudan, M., Barrington, C., Henderson, S., Ernst, C., Odom, D.T., Tanay, A., and Hadjur, S. (2015). Comparative Hi-C reveals that CTCF underlies evolution of chromosomal domain architecture. Cell Rep. *10*, 1297–1309.

Wei, Z., Gao, F., Kim, S., Yang, H., Lyu, J., An, W., Wang, K., and Lu, W. (2013). Klf4 organizes long-range chromosomal interactions with the oct4 locus in reprogramming and pluripotency. Cell Stem Cell *13*, 36–47.

Wendt, K.S., Yoshida, K., Itoh, T., Bando, M., Koch, B., Schirghuber, E., Tsutsumi, S., Nagae, G., Ishihara, K., Mishiro, T., et al. (2008). Cohesin mediates transcriptional insulation by CCCTC-binding factor. Nature *451*, 796–801.

de Wit, E., Bouwman, B.A., Zhu, Y., Klous, P., Splinter, E., Verstegen, M.J., Krijger, P.H., Festuccia, N., Nora, E.P., Welling, M., et al. (2013). The pluripotent genome in three dimensions is shaped around pluripotency factors. Nature *501*, 227–231.

de Wit, E., Vos, E.S.M., Holwerda, S.J.B., Valdes-Quezada, C., Verstegen, M.J.A.M., Teunissen, H., Splinter, E., Wijchers, P.J., Krijger, P.H.L., and de Laat, W. (2015). CTCF binding polarity determines chromatin looping. Mol. Cell *60*, 676–684.

Wray, J., Kalkan, T., Gomez-Lopez, S., Eckardt, D., Cook, A., Kemler, R., and Smith, A. (2011). Inhibition of glycogen synthase kinase-3 alleviates Tcf3

repression of the pluripotency network and increases embryonic stem cell resistance to differentiation. Nat. Cell Biol. *13*, 838–845.

Xiang, W., Roberti, M.J., Hériché, J.K., Huet, S., Alexander, S., and Ellenberg, J. (2018). Correlative live and super-resolution imaging reveals the dynamic structure of replication domains. J. Cell Biol. *217*, 1973–1984.

Yaffe, E., and Tanay, A. (2011). Probabilistic modeling of Hi-C contact maps eliminates systematic biases to characterize global chromosomal architecture. Nat. Genet. *43*, 1059–1065.

Ying, Q.L., Stavridis, M., Griffiths, D., Li, M., and Smith, A. (2003). Conversion of embryonic stem cells into neuroectodermal precursors in adherent monoculture. Nat. Biotechnol. *21*, 183–186.

Ying, Q.L., Wray, J., Nichols, J., Batlle-Morera, L., Doble, B., Woodgett, J., Cohen, P., and Smith, A. (2008). The ground state of embryonic stem cell self-renewal. Nature *453*, 519–523.

Zessin, P.J.M., Finan, K., and Heilemann, M. (2012). Super-resolution fluorescence imaging of chromosomal DNA. J. Struct. Biol. *177*, 344–348.

Zhan, Y., Mariani, L., Barozzi, I., Schulz, E.G., Blüthgen, N., Stadler, M., Tiana, G., and Giorgetti, L. (2017). Reciprocal insulation analysis of Hi-C data shows that TADs represent a functionally but not structurally privileged scale in the hierarchical folding of chromosomes. Genome Res. *27*, 479–490.

Zhang, Y., Liu, T., Meyer, C.A., Eeckhoute, J., Johnson, D.S., Bernstein, B.E., Nusbaum, C., Myers, R.M., Brown, M., Li, W., et al. (2008). Model-based analysis of ChIP-Seq (MACS). Genome Biol. *9*, R137.

STAR★METHODS

KEY RESOURCES TABLE

REAGENT or RESOURCE	SOURCE	IDENTIFIER
Antibodies		
Anti-CTCF	Milipore	Cat# 07-729; RRID: AB_441965
Anti-Nestin	Developmental Studies Hybridoma Bank	Cat# Rat-401; RRID: AB_2235915
Anti-Oct3/4	Santa Cruz Biotechnology	Cat# sc-5279; RRID: AB_628051
Anti-Rad21	Abcam	Cat# ab-992; AB_2176601
Anti-mouse Alexa Fluor 532	Invitrogen	Cat# A11002; RRID: AB_2534070
Phycoerythrin-labelled secondary antibody	Santa Cruz Biotechnology	Cat# sc-3761; RRID: AB_639241
Chemicals, Peptides, and Recombinant Proteins		
DMEM/F-12	Thermo Fisher	31331028
GMEM	Invitrogen	11710035
N2 supplement	Thermo Fisher	17502-048
B27 supplement	Thermo Fisher	17504-04
Non-essential amino acids	Thermo Fisher	11140050
L-glutamine	Thermo Fisher	25030081
Bovine serum albumin (BSA, fraction V)	Thermo Fisher	15260037
CHIR99021 (GSK3ß inhibitor)	Trevigen (Reagents Direct)	27-H76
PD0325901 (MEK inhibitor)	Trevigen (Reagents Direct)	PD0325901
Leukemia Inhibitory Factor (LIF)	This study	This study
Epidermal Growth Factor (EGF)	This study	This study
Basic Fibroblast Growth Factor (bFGF)	This study	This study
Activin A	Sigma	A4941
Fibronectin	Sigma	F1141
Laminin	Sigma	L2020
Accutase	Sigma	A6964-100ML
T1 paramagnetic beads	Thermo Fisher	65602
C1 paramagnetic beads	Thermo Fisher	65002
Ampure XP paramagnetic beads	Beckman Coulter	A63881
Protein A Dynabeads	Invitrogen	10002D
Complete Mini EDTA-free proteinase inhibitor	Roche	11836170001
Catalase	Sigma	C3155
Glucose oxidase	Sigma	G0543
HindIII	New England Biolabs	R0104S
MboI	New England Biolabs	R0147S
Quick Ligation kit	New England Biolabs	M2200S
T4 DNA ligase	New England Biolabs	M0202S
Exonuclease III (*E. coli*)	New England Biolabs	M0206S
Klenow Fragment (3'→5' exo-)	New England Biolabs	M0212S
DNA Polymerase I, Large (Klenow) Fragment	New England Biolabs	M0210S
T4 Polynucleotide Kinase	New England Biolabs	M0201S
T4 DNA polymerase	New England Biolabs	M0203S
Phusion 2x master mix	New England Biolabs	M0536S
EZlink Iodoacetyl-PEG2-Biotin	Thermo Fisher	21334
NEXTFlex DNA Barcodes	Bioo Scientific	NOVA-514102
2′-Deoxyguanosine-5′-O-(1-thiotriphosphate) sodium salt, Sp-isomer	Axxora	BLG-D031-05

(*Continued on next page*)

Continued

REAGENT or RESOURCE	SOURCE	IDENTIFIER
EdU	Molecular Probes	C10340
Alexa Fluor 647	Molecular Probes	C10340
Slide-A-lyzer	Thermo Fisher	66003
Critical Commercial Assays		
RNase A	Thermo Fisher	EN0531
Proteinase K	New England Biolabs	P8107S
Ovation Ultralow library system V2	NuGEN	034432
TRIzol reagent	Thermo Fisher	15596026
TURBO DNase	Ambion	AM2238
Agilent 2100 Bioanalyzer RNA 6000 Nano	Agilent	5067-1511
TruSeq Stranded mRNA Sample Prep Kit	Illumina	20020594
Deposited Data		
TCC	https://www.ebi.ac.uk/arrayexpress	E-MTAB-2063
In-situ Hi-C	https://www.ebi.ac.uk/arrayexpress	E-MTAB-6591
ChIP-seq	https://www.ebi.ac.uk/arrayexpress	E-MTAB-5732
RNA-seq	https://www.ebi.ac.uk/arrayexpress	E-MTAB-2125
Experimental Models: Cell Lines		
Sox1-GFP mouse embryonic stem cells (46C)	Austin Smith	12524553
Neural stem cells	This study	This study
Post-implantation epiblast stem cells (EpiSC)	This study	This study
ESC line with stably inserted FUCCI system	Matthias Lutolf	23193167
Experimental Models: Organisms/Strains		
Mus musculus 129P2/Ola	NA	NA
Software and Algorithms		
Leica SR GSD Wizard	https://www.leica-microsystems.com	NA
MATLAB 2012b	https://www.mathworks.com/downloads/web_downloads/select_release	NA
Juicer	https://github.com/theaidenlab/juicer/wiki	NA
Juicebox v1.0	https://github.com/theaidenlab/juicebox/wiki	NA
SAMtools 0.1.19	http://samtools.sourceforge.net/	NA
BEDtools	http://bedtools.readthedocs.io/en/latest/	NA
Bowtie 2	http://bowtie-bio.sourceforge.net/bowtie2/index.shtml	NA
TopHat 2	https://ccb.jhu.edu/software/tophat/manual.shtml	NA
MACS 1.4	https://github.com/downloads/taoliu/MACS/macs_1.4.2.deb	NA
MACS 2	http://liulab.dfci.harvard.edu/MACS/	
R 3.2.2	https://cran.r-project.org/src/base/R-3/R-3.2.2.tar.gz	NA
edgeR_3.12.1	https://www.bioconductor.org	NA
DESeq2_1.10.1	https://www.bioconductor.org	NA
DESeq_1.22.1	https://www.bioconductor.org	NA
ggplot2_2.1.0	https://www.bioconductor.org	NA
Matrix_1.2-6	https://www.bioconductor.org	NA
IRanges_2.4.8	https://www.bioconductor.org	NA
GenomicRanges v1.22.4	https://www.bioconductor.org	NA
Analysis code and vignette	This study	NA
Reanalyzed Data		
H3K4me1 (ESCs)	https://www.ebi.ac.uk/ena	SRP000230
H3K4me1 (NP cells)	https://www.ebi.ac.uk/ena	SRP000230

(*Continued on next page*)

Continued

REAGENT or RESOURCE	SOURCE	IDENTIFIER
H3K4me3 (ESCs)	https://www.ebi.ac.uk/ena	SRP000230
H3K4me3 (NP cells)	https://www.ebi.ac.uk/ena	SRP000230
H3K27ac (ESCs)	https://www.ebi.ac.uk/ena	SRP003638
H3K27ac (NP cells)	https://www.ebi.ac.uk/ena	SRP003638
GNF Mouse GeneAtlas V3	https://www.ncbi.nlm.nih.gov/geo	GSE10246
Expression data from murine NP cells	https://www.ncbi.nlm.nih.gov/geo	GSM198065, GSM198066, GSM198067
Genomic coordinates of enhancer elements identified in brain tissues	chromosome.sdsc.edu/mouse/download.html	NA
Hi-C data (NS and NSC derived iPS cells [passage 20])	https://www.ncbi.nlm.nih.gov/geo	GSE76479

CONTACT FOR REAGENT AND RESOURCE SHARING

Further information and requests for resources and reagents should be directed to and will be fulfilled by the Lead Contact, Wolfgang Huber (whuber@embl.de).

EXPERIMENTAL MODEL AND SUBJECT DETAILS

Cell Culture Conditions

Pluripotent Stem Cell Culture

ES Cell Culture in Standard Conditions (FBS/LIF). Sox1-GFP mouse embryonic stem (ES) cells derived from the E14tg2a line (46C, (Ying et al., 2003)) were grown at 37°C in a 5% (v/v) CO2 incubator in Glasgow Minimum Essential Medium (GMEM, Invitrogen) supplemented with 10% (v/v) fetal bovine serum (FBS, Sigma), 2 ng/ml leukemia inhibitory factor (LIF, in-house), 1 mM 2-mercaptoethanol, non-essential amino acids (Gibco), L-glutamine (Gibco) and Na-pyruvate (Gibco), on gelatin-coated (0.1% v/v) plates. Accutase (Sigma) was used for cell dissociation. Cells were passaged every second day and seeded at a density of 1.3 million cells per 10 cm surface area of the corresponding culture vessel. Medium was exchanged daily.

ES Cell Culture in Chemically Defined Conditions (2i/LIF). ES cells were grown at 37°C in a 5% (v/v) CO2 on 0.1% (v/v) gelatin-coated flasks in the presence of GSK3ß and MEK inhibitors plus LIF (2i/LIF). Complete medium comprised 50% Dulbecco's Modified Eagle's Medium (DMEM), 50% F12 (DMEM/F-12, Invitrogen), supplemented with 2.5 ml of N2 and 5ml of B27 (Gibco), bovine serum albumin (BSA) fraction V (Gibco; final concentration 0.012%), non-essential amino acids (Gibco), glucose (final concentration 0.03 M), HEPES (final concentration 4.5 mM) and 0.1 mM beta-mercaptoethanol, supplemented with GSK3ß inhibitor CHIR99021 (Trevigen) at a final concentration of 3 μM, MEK inhibitor PD0325901 (Trevigen) at a final concentration of 1 μM, and 2 ng/ml LIF.

EpiSC Differentiation and Culture. ES cells were transferred to culture vessels coated overnight with fibronectin (1:60, Sigma) freshly diluted in phosphate buffered saline (PBS). Cells were maintained in N2+B27 supplemented medium described above, with 20 ng/ml Activin A and bFGF (in-house) at a final concentration of 12 ng/ml. Cultures were propagated for a minimum of eight passages to establish an EpiSC identity.

NS Cell Differentiation and Culture. ES cells were plated at a density of 0.8 million cells per 10 cm gelatin-coated culture vessels in neural differentiation medium comprising 50% DMEM and 50% F12 (DMEM/F-12, Invitrogen) supplemented with 2.5 ml N2 and 5 ml of B27 (Gibco), BSA (Gibco, final concentration 0.012%), non-essential amino acids (Gibco), glucose (final concentration 0.03 M), HEPES (final concentration 4.5 mM) and 0.1 mM beta-mercaptoethanol. Medium was exchanged after 24 and 48 hours, and cultures were grown for an additional 72 hours. Cells were then dissociated using Accutase (Sigma) and the GFP+ fraction (ca. 70% of cells) was sorted by flow cytometry and seeded into a laminin (Sigma) coated 75 cm^2 flask (10 $\mu g/cm^2$ laminin, minimum 4 h coating time at 37°C). Subsequently, cells were grown in neural diafferentiation medium supplemented with recombinant murine EGF and bFGF (in-house; final concentration 10 ng/ml) until loss of GFP expression and uniform upregulation of Nestin was observed by immunostaining and qRT-PCR. NS cells were passaged at 80% confluence. Medium was exchanged daily.

METHOD DETAILS

Analysis by Flow Cytometry

Cells were fixed in 4% paraformaldehyde (Sigma) for 10 minutes at room temperature. Cells were spun for 2 min at 400 × g at 4°C and washed 2 times with ice cold PBS, then permeabilized by incubation in 0.1% Triton-X100 solution in PBS for 20 min on ice. After 2 washes with PBS, cells were incubated in blocking solution comprising PBS supplemented with 10% FBS for 2 h on ice. Anti-Oct4

(sc-5279, Santa Cruz, final dilution 1:200) or anti-Nestin (Rat-401, HSHB, 1:100) were added and cells were incubated with gentle shaking for 2 h. Cells were processed 3 times in a washing solution of 1% BSA and 0.025% TritonX-100 in PBS and resuspended in blocking solution supplemented with phycoerythrin-labelled secondary antibody (sc-3761, Santa Cruz, 1:400). After 45 min incubation at 4°C, cells were washed twice in ice cold washing solution, resuspended in PBS and analyzed with a FACScan flow cytometer.

ChIP-Seq

Cells were detached from culture vessels with Accutase dissociation reagent (Sigma). After washing with PBS at room temperature (RT), 25 million cells were resuspended in 25 ml culture medium and formaldehyde (Sigma) was added to a final concentration of 1%. Cells were incubated at RT for 10 min with occasional mixing. Following the incubation, glycine was added to a final concentration of 0.125M to quench the reaction, and the cell solution was incubated at RT for 5 min followed by 5-min incubation on ice. Cells were spun for 10 minutes at 300 × g at 4°C and washed twice with ice cold PBS. Pellets were snap frozen on dry ice and stored at -80°C. On the day of the experiment, crosslinked cell pellets were thawed on ice for 10 minutes. Lysis buffer was added to a final volume of 1 ml (10 mM Tris pH 7.5, 1 mM EDTA, 0.1% SDS, 0.1% sodium deoxycholate, 1% Triton X-100, 1× Complete Mini EDTA-free proteinase inhibitor (Roche)). Cells were incubated on ice for 20 minutes. Chromatin extracts were sheared with a Branson sonification instrument using 18 cycles of 20 s sonication at 35% amplitude followed by a 30 s pause, maintaining samples on ice. After chromatin sonification a 25 µl aliquot was incubated with 1 µl RNase A for 30 min at 37°C. Next, 1 µl of Proteinase K (NEB) was added along with SDS to a final concentration of 1%. Samples were incubated for 1 h at 55°C. Extracts were then incubated at 95°C for 2 min. DNA was precipitated with 12 µl of 3M sodium acetate and 120 µl pure ethanol on ice for 10 min. Samples were spun at 14,000 × g at 4°C. Pellets were washed with 75% ice-cold ethanol. DNA concentration was evaluated on the NanoDrop spectrophotometer. Chromatin extracts corresponding to 10 M cells (90 µg DNA) were resuspended in a total volume of 450 µl lysis buffer and 40 µl Protein A Dynabeads (Thermo Scientific), previously washed once with PBS, were added. Samples were incubated at 4°C with overhead mixing for 1 h to pre-clear the chromatin. During this time, Protein A Dynabeads were washed with PBS and incubated with anti-CTCF (Milipore, 07-729) or anti-Rad21 (Abcam, ab-992) at RT with overhead mixing (40 µl beads per 5 µl anti-CTCF or 5 µg anti-Rad21 was used). Beads were washed with PBS. Finally, 500 µl of pre-cleared chromatin was mixed with antibody-coupled beads and incubated overnight at 4°C with overhead mixing. The following day, the beads were washed for 10 min at 4°C with overhead mixing in the following buffers: lysis buffer (twice), lysis buffer containing 0.3M NaCl (twice), LiCl buffer (0.25 M LiCl, 0.5% IGEPAL-630, 0.5% sodium deoxycholate, twice), TE (pH 8.0) plus 0.2% Triton X-100 (once), and TE (pH 8.0, once). After the final wash, beads were resuspended in 100 µl TE (pH 8) and incubated at 65°C for 14 h. Next, 1 µl RNase A was added and the beads incubated for 1 h at 37°C. Following this step, 10 µl Proteinase K was added and the sample incubated at 55°C for 2 h. Beads were separated from the solution using a magnet. DNA was purified from 110 µl of the above solution with 200 µl of Ampure XP paramagnetic beads (Beckman Coulter). Sequencing libraries were prepared with the Ovation Ultralow library system V2 (NuGen) using 1/5 of the sample material (2 ng). Libraries were sequenced on the Illumina HiSeq 3000 in 50 bp single-end mode.

Tethered Chromatin Conformation

TCC analysis was performed with HindIII on 25 million cells as previously described (Kalhor et al., 2011). Libraries were sequenced on the Illumina HiSeq 2000 in 50 bp paired-end mode.

In-Situ Hi-C

In-situ Hi-C was performed with MboI as previously described (Rao et al., 2014). Two pellets of 5 million crosslinked cells were processed for each sample. Libraries were sequenced on the Illumina NextSeq 500 in 80 bp paired-end mode.

RNA-Seq

Cells were dissociated with Accutase (Sigma), washed twice in basal medium, and lysed in TRIzol reagent (Invitrogen, 500 µl per 5 million cells). Phase extraction was performed according to the standard protocol. RNA samples were treated with TURBO DNase (Ambion) and purified according to the manufacturer's instructions. RNA quality was assessed on the Agilent 2100 Bioanalyzer with the RNA 6000 Nano assay. Samples with RNA Integrity Number (RIN) greater than 9 were included in subsequent experiments. Polyadenylated transcripts were isolated from 3 µg total RNA by oligo-dT magnetic beads, and sequencing libraries were produced using the TruSeq Stranded mRNA Sample Prep Kit (Illumina).

Super-Resolution Imaging

EdU Labelling

FUCCI ES cells (FBS/LIF) were pulsed for 5 s with 100µM EdU (Molecular Probes) in PBS, rinsed 3 times and sorted by flow cytometry to enrich for cells in early S phase. Half of the recovered cells were then seeded in ES cell medium (FBS/LIF) and the other half in neural differentiation medium.

Click Chemistry and Immunofluorescence

ES and NS cells were cultured on gelatin- or laminin-coated Labtek chambers (Nunc, 734-2062), respectively. Cells were fixed for 15 min with 3.7% paraformaldehyde (EMS, 15710) in PBS at RT, and washed 3 times for 5 min with 3% BSA in PBS. Samples were permeabilized for 20 min at RT with 0.2% Triton X100 in PBS, washed 3 times for 5 min with 3% BSA in PBS, and processed

for the click chemistry reaction with Alexa Fluor 647 as described by the vendor (Molecular Probes). Samples were washed 3 times for 5 min with 3% BSA in PBS and incubated with anti-Nestin antibody (DSHB, rat-401, 1:50) for 1 h at 4°C. Samples were washed twice for 5 min with 0.01% Tween and 1% BSA in PBS, and incubated with an anti-mouse Alexa Fluor 532 conjugate (2 μg/ml, Invitrogen) for 1 h at RT. Samples were washed and stored in PBS for imaging.

Image Acquisition

Super-resolution microscopy was performed on the Leica SR 3D Ground State Depletion (GSD) instrument. Cells were maintained in blinking buffer containing 10 mM MEA/GLOX, 50 mM Tris pH 8.5, 10 mM NaCl, 10% w/v glucose, 0.5 mg/ml glucose oxidase (Sigma), 40 μg/ml catalase (Sigma), 10 mM MEA (stock at -20°C; 100 mM in 1x PBS and pH 7.4, titrated with HCl). Fresh buffer was prepared immediately prior to each imaging series. Buffer was changed every 2–3 h. GSD images were acquired with a 642 nm laser (500 mW) and a 405 nm diode laser (30 mW) on a Coherent Inc. and Suppressed Motion (SuMo) stage. Objectives used were Leica HCX PL APO 100x, NA 1.47 Oil CORR TIRF PIFOC and HCX PL APO 160x, 1.43 Oil CORR TIRF PIFOC. Images were acquired with an Andor iXon3 897 EMCCD camera at 100 nm resolution. The microscope was operated in epifluorescence mode. The system was left to equilibrate for 2 h before system start and sample mounting. Samples were exposed to maximum laser power at 642 nm until single-fluorophore blinking was detected. Thereafter a UV-405 nm laser was used for back pumping. Series of at least 30,000 frames were acquired.

Image Processing

Single-molecule events in all GSDIM movies were identified with the Leica SR GSD Wizard, setting a photon threshold of 25. The resulting event list was imported and further processed in MATLAB. Locations with fewer than 500 photons were excluded. Lateral drift was corrected using a correlation-based algorithm (Szymborska et al., 2013). Image resolution was estimated at ca. 30 nm by Fourier ring correlation (Banterle et al., 2013). A median filter (3×3 neighborhood) was applied to the reconstructed raw images. Pixels not connected to at least 3 others were excluded. Replication forks were detected by grayscale dilation as follows: a disc of radius 4 pixels was used as a structural element. For each pixel in the image, the highest intensity in the neighborhood defined by the structural element was determined. This yielded a grayscale dilated image that was subtracted from the original. Local maxima have intensity value 0 in the subtracted image. When directly neighboring pixels were simultaneously identified as local maxima, only one was retained for analysis. Identified forks were subjected to nearest neighbor analysis, estimating the mean nearest neighbor distance (NND) and plotting the distribution in each condition.

QUANTIFICATION AND STATISTICAL ANALYSIS

High-Throughput Sequencing Data Analysis

Chromatin Conformation Data

TCC and In Situ Hi-C Data Pre-processing. Both TCC and Hi-C data were preprocessed with Juicer using default settings (Durand et al., 2016a; Rao et al., 2014). Reads were mapped to the *Mus musculus* MGSCv37 (mm9) genome assembly, where libraries from each technical replicate were processed separately. After this step, files from the two technical replicates were merged, sorted and .hic files were obtained using the pre function from Juicer. Matrices 5, 10 and 50kb resolution were extracted from .hic files using the dump command from Juicer and processed in R as indicated below.

TCC and In-Situ Hi-C Data Normalization. We used Iterative Proportional Fitting (IPF, (Imakaev et al., 2012; Pukelsheim and Simeone, 2009; Rao et al., 2014)) to account for the dependence of ligation frequency matrices upon not only physical proximity between sequences in the interphase nucleus, but also on biases introduced by chromatin accessibility, restriction fragment length and composition (Imakaev et al., 2012; Yaffe and Tanay, 2011). These factors impart differences in the overall visibility of each genomic interval (TCC: 10 kb bin, in-situ-Hi-C: 5kb) in the TCC assay. In IPF, biases are inferred from the coverage for each bin and parameterized in a bias coefficient. We applied the version of IPF suggested by Pukelsheim and Simeone, with 20 iterations. Here, let indices i, j denote two bins, b_i the IPF-derived bias coefficient for bin i, then the IPF-normalized count is

$$N_{ij} = C_{ij} \big/ (b_i\, b_j).$$

For visualization purposes, normalized matrices were exported to .txt files which were then used to assemble .hic files, using the pre function form Juicer. We used Juicebox (Durand et al., 2016b) to visualize the data.

Calling Loops from TCC Data

We implemented a two-step process for loop detection from TCC data. First we identified interactions. Then we called loop instances, taking into account local clustering of significant interactions as detailed below.

Identification of Significant Interactions

We defined interactions as bin pairs on the same chromosome with IPF-normalized counts significantly higher than those typically observed at the same genomic distance. Interaction calling entailed three steps: estimation of the expected IPF-normalized count due to genomic distance alone, local signal aggregation, and statistical testing for significance of a given interaction, considering both the expected value from the distance fit and the statistical variability (technical and biological) in the data.

Interaction Calling: Estimation of Genomic Distance Effect

For each chromosome, we fit a distance-dependence function $E(r)$, expressing the expected IPF-normalized counts between bins separated by genomic distance r, by averaging, for each r, the IPF-normalized counts N_{ij} for all bin pairs separated by distance r,

and smoothing the resulting function of r with a running average filter with window half-size 1.5 bins. We defined the expected count S_{ij} for bin pair *i, j* as $S_{ij} = b_i\, b_j\, E(r)$ (note that S_{ij} is not required to be an integer).

Interaction Calling: Initial Filtering and Local Signal Aggregation

We identified a set of 10 kb bin pairs with at least one ligation product in both biological replicates and termed these putative interactions. We next considered the 3×3 square of pixels B centered on a putative interaction between bins i and j, where

$$B = \{k, l| \quad |k - i| + |l - j| \quad <= 2\},$$

and computed a local aggregate

$$C'_{ij} = \sum_{k,l,\in B} C_{kl},$$

as well as for the expected counts (S'_{kl})

$$S'_{ij} = \sum_{k,l,\in B} S_{kl}.$$

Note that these values are still interpretable as sequencing counts (i.e., there was no division by |B|=9 as there would be for a local average). They served as input to the statistical testing procedure described below.

Interaction Calling: Statistical Testing

We tested bin pairs on the same chromosome, and within 2 Mb genomic distance. To determine whether raw counts (C'_{ij}) for a bin pair were significantly higher than the expected counts (S'_{ij}) in both replicates, we used a statistical model based on the Gamma-Poisson (negative binomial) distribution, as established in the analysis of count data from high-throughput sequencing (Love et al., 2014). We computed an estimate of the dispersions with the edgeR package (Robinson et al., 2010) in “classic mode” on sample replicates using log(S'_{ij}) values as offsets, and with the moving average option. Note that edgeR does not use offsets in classic mode directly, such that the dispersion estimate is based on raw counts C'_{ij}. This estimate is conservative (biased upwards), which helps to avoid false-positive results in the statistical testing step.

We then provided estimated dispersions as well as S'_{ij} as normalization factors to DESeq2 (Love et al., 2014) and fit an intercept-only model with beta-shrinkage disabled. We interpreted the intercept coefficients β_{ij} as a measure of evidence for a specific interaction between bins *i* and *j*. We performed a one-sided Wald test on these coefficients, under a null hypothesis $\beta_{ij} \leq 0$. We disabled independent filtering and outlier detection options in DESeq2, so that we obtained a p-value for each bin pair. We then applied the Benjamini-Hochberg method for false discovery rate (FDR) control in multiple testing and considered interactions with an adjusted p-value less than 20% and fold change >1.5 as significant.

Identification of Chromatin Loops from TCC Data

We identified regions exhibiting strong local signal indicating loop structures by adopting a local background approach (Rao et al., 2014). To this end, it is useful to treat a 2-dimensional array of normalized counts N_{ij} as an image and each bin pair a pixel. The loop detection method is described in Figure S1D. It is important to note that the design of this approach identifies only those loops for which the two bins (which we also term “anchors”) are separated by at least 120 kb. Coordinate bin pairs corresponding to pixels forming a loop were extended by 10 kb in both directions and considered loop anchors. We observed frequent clustering of loop anchors whereby two consecutive genomic bins were identified as anchors of the same loop. To enumerate loops in each condition, we merged such cases and counted them as a single instance.

Calling Loops from In-Situ Hi-C Data

We pooled the filtered unique Hi-C interactions from the two biological replicates (merged_nodups.txt files generated by Juicer), then sorted them via the standard command-line function (-m –k2,2d –k6,6d) along with the .hic file generated by the pre command from Juicer (pre –q 30 sorted_merged_nodups.txt merged.hic mm9.) We then used HiCUPPS to call loops (Rao et al., 2014) as follows: juicebox hiccups -m 1024 -r 5000,10000 -k KR -f 0.1 -p 4 -i 10 -t 0.01,1.5,1.75,2 inter30.hic path/to/loop/files.

Testing for Differences in Loop Strength

Our approach for quantitative comparison of loop strength followed the approach outlined in Identification of significant interactions. We first produced aggregated raw (C'_{ij}) and expected (S'_{ij}) counts for each loop identified in the cell types assayed by summation, analogous to the procedure detailed in Identification of significant interactions (interaction area in the TCC data: 9 pixels (resolution of 10kb); interaction area in the Hi-C data: 9 pixels (resolution of 5kb)). Following this initial aggregation step, overlapping interaction areas were merged.

For each of the two comparisons of loops identified from TCC data: ES (FBS/LIF) vs. NS cells (Figures S1C and S1F), ES (2i/LIF) vs. NS cells (Figure 5)) and in the in-situ Hi-C data, we analyzed loops identified in either or both conditions. In the comparison between ES cell (2i/LIF) and EpiSC, we considered all the loops identified in ES (2i/LIF) and NS cells (TCC data, Figure 5).

We then computed an estimate of the dispersions on the four samples with the edgeR (Robinson et al., 2010) package in classic mode. We considered log(S'_{ij}) as offsets, used the moving average option and provided the experimental groups in the model. Next, we provided the estimated dispersions as well as the expected counts S'_{ij} as normalization factors to DESeq2 (Love et al., 2014). We fit a model including a coefficient γ_{ij} that represents the log2 fold change with beta-shrinkage disabled. We interpreted the intercept coefficients γ_{ij} as a measure of evidence for differential loop formation. We performed a two-sided Wald test on these coefficients,

assuming a null hypothesis $|\gamma_{ij}| = 0$. We disabled independent filtering and outlier detection in DESeq2, so that we obtained a p-value for each interaction area. We applied the Benjamini-Hochberg multiple testing to correct the p-values.

Computation of the Directionality Index

Following an approach adapted from (Dixon et al., 2012), we inferred the boundaries of TADs by calculating the directionality index (DI), a χ-square-like statistic that indicates whether each 10 kb bin interacts predominately with bins to its left or right, and defined:

$$\mathrm{DI}_i = \mathrm{sign}(B_i - A_i)\left(\frac{(A_i - E_i)^2}{E_i} + \frac{(B_i - E_i)^2}{E_i}\right) = \mathrm{sign}(B_i - A_i)\left(\frac{(A_i - B_i)^2}{(A_i + B_i)}\right),$$

where A_i is the sum of the normalized signal (N_{ij}) within a window of 2 Mb to the left of the bin i, B_i the corresponding quantity for a window to the right, and $E_i = (A_i + B_i) / 2$ estimates the normalized signal (N_{ij}) to either side that would be expected in the absence of directionality.

Identification of A and B Compartments

We partitioned the genome into open/active and closed/inactive compartments, termed A and B respectively, according to (Kalhor et al., 2011; Lieberman-Aiden et al., 2009). For each chromosome, we considered the normalized intra-chromosomal interaction matrix W at 50kb resolution (in-situ Hi-C data, Figure S2H). An overall distance-dependent trend $d(\ |i\text{-}j|\ )$ was fit onto the coverage-normalized W as follows: we first computed at each genomic distance (off diagonal in W) the mode value, then for each genomic distance we estimated a smoothened value of the mode using the smooth.spline function in R. We then computed the overall normalized matrix $N_{ij} = W / d(\ |i\text{-}j|\ )$. For each chromosome, we computed the first three eigenvectors (corresponding to the three largest eigenvalues) of the correlation matrix of N_{ij}. In each sample and each chromosome, we considered the eigenvector value that captured best the partition of the normalized interaction matrix into two blocks of interactions (W, (Kalhor et al., 2011)). Then for each chromosome, we separated the bins with positive and negative sign of the eigenvector.

We compared gene expression levels in the two groups. The group containing genes with higher aggregate expression was labelled cluster A, the other cluster B. The eigenvector signs were also fixed to reflect this separation, where eigenvectors for bins in cluster A were assigned positive values and those for bins in cluster B were set to negative.

To annotate loop domains to compartments (Figure S2H), for each domain we computed the percentage of bins at 50 kb resolution within the domain assigned to compartment A. If this value exceeded 50%, the domain was considered to be within compartment A.

Identification of Contact Domains

We pooled reads from the two biological replicates (as described in Calling loops from in-situ Hi-C data). Contact domains (CD) were identified using the arrowhead function from juicer with default parameters (arrowhead -m 2000 -r 10000 -k KR).

For each CD, start and end coordinates were considered separately. Genomic positions were extended by 15 kb in both directions to obtain 30k domain boundary intervals.

CTCF peaks overlapping the domain boundary intervals defined above were considered in Figure 4E. Peaks in the "other locations" class comprised CTCF peaks that did not map within the extended boundary regions (domain boundary intervals +/- additional 35 kb). The normalized ratio of CTCF and Rad21 signals between NS and ES (FBS/LIF) was computed with DESeq2.

Loop Domains and Ordinary Domains

We defined loop and ordinary domains based on the overlap between loop anchors and domain boundaries. The coordinates of 5′ and 3′ loop anchors were extended by 20kb in both directions. In parallel, the coordinates of domain boundaries were also extended by 20kb in both directions. Loop domains were defined as the CD for which 5′ and 3′ boundaries intersected the two anchors of the same loop. Ordinary domains were defined as domains that did not contain an internal loop and that were not within loop domains themselves.

Analysis of Chromatin Contact Insulation

As illustrated in Figure 4A, insulation of a genomic region (here a 10 kb bin) reflects the number of interactions crossing a bin relative to the average number of interactions in the two neighboring domains it separates (Sofueva et al., 2013). We thus defined an insulation score as the ratio of the average interaction strength at both sides of the bin (inside) to the interaction strength across the bin (between).

For a given bin B, we set a distance Z at which the insulation effect is to be assessed (see below for discussion). The distance Z in the interaction matrix is the off-diagonal at which we test the insulation effect of a bin, where Z=5. For each bin B we define a pixel $X'_{i,j}$, where $i = B - Z \wedge j = B + Z$. TCC (or Hi-C) signal at this pixel corresponds to the number of interactions crossing the bin at distance Z (interaction strength depicted as between in Figure 4A). To reduce the noise, we summed the signal in a square centered on the pixel $X'_{i,j}$ and of size equal to 25 pixels (5×5 bins).

$$X'_{ij} = \sum_{k,l,\in A} S_{kl},$$

where

$$A = \{k,l \,|\, |k - i| + |l - j| \le 4\}.$$

To estimate the expected local interaction strength, we translated the 5×5 pixel square by N pixels in the 5′ (left aggregate, $L'_{iL,jL}$) and 3′ (right aggregate, $R'_{iR,jR}$) directions, and summed the normalized interaction signal in both. This operation did not change the genomic distance between bins within the square, only the position relative to bin B.

Left ($L'_{iL,jL}$) and right ($R'_{iR,jR}$) aggregates were defined as follows:

$$L'_{iL,jL} = \sum_{k,l,\in E} S_{kl} \ \ and \ \ R'_{iR,jR} = \sum_{k,l,\in G} S_{kl}$$

$$E = \{k,l||k-i|+|l-j|\leq 4\}$$

$$G = \{k,l||k-i|+|l-j|\leq 4\}$$

$$iL = i - D \wedge jL = j - D \wedge iR = i + D \wedge jR = j + D.$$

We computed the average of the two expected values and divided the result by the normalized number of interactions crossing the bin obtained in the first step. We then considered the logarithm of this value.

The insulation score (IS) is thus defined as follows:

$$IS_{ij} = \log_2\left(\frac{\left(L'_{i-D,j-D} + R'_{i+D,j+D}\right)\cdot 0.5}{X'_{ij}}\right),$$

Where:

X'_{ij} = sum of the normalized TCC signal in the central square

D = distance (in the number of 10 kb bins) between midpoints of the central and left squares, and between the right and central squares

L'_{ij} = sum of the normalized TCC signal in the left square

R'_{ij} = sum of the normalized TCC signal in the right squarel

S_{ij} = insulation score at pixel of coordinates i and j corresponding to the midpoint of the central square.

To facilitate this analysis on all loop anchors required an optimal D. This parameter must be set in such a way that it will capture the contact insulation effect exerted by a bin, without being skewed by the presence of a loop or the boundary of a TAD in the local neighborhood. As depicted in the composite TCC profile in Figure 5F, loops produce a visual stripe of strong signal around 5 pixels wide, emanating from the anchor towards the centre of the domain. Inclusion of this signal would artificially enhance the insulation score. Conversely, D should not be too large as the resulting square might exit the contact domains separated by the bin under consideration and become situated in the inter-domain space. This in turn would artificially reduce the insulation effect.

We found that D of 15×10 kb bins optimally captured this effect. Figure 4B compares the insulation of $CTCF^+$ bins within TAD boundaries. $CTCF^+$ bins were defined as those intersecting a CTCF peak and a TAD boundary (TAD boundary coordinates were extended by ±10 kb for this comparison). These bins were further stratified into two classes based on the overlap between loop anchors and TAD boundaries. $Loop^+$ bins denote $CTCF^+$ bins within TAD boundaries that overlap a loop anchor. Loop- bins indicate $CTCF^+$ bins within TAD boundaries that did not overlap a loop anchor (loop anchors were extended by ±10 kb prior to this computation).

In Figure 4C we considered anchors of dynamic and common loops (identified through the comparison between in-situ Hi-C profiles of ES and NS cells: dynamic loops with FC>1.5 and adjusted p<0.1; common loops were instances with FC<1.25).

In each IS calculation in Figure 4, we considered pooled normalized matrices at 10 kb resolution.

Analysis of Public Domain In Situ Hi-C Data

Pre-processed in-situ Hi-C data from ES and NS cells, and from iPS cells derived from NS cells (passage 20) (Krijger et al., 2016) were obtained from GEO (see Key Resources Table) and binned at 10kb resolution. These data were normalized as described above.

ChIP-Seq Data Analysis

ChIP-seq data were either generated as part of this study (CTCF and Rad21) or obtained from the European Nucleotide Archive (Key Resources Table).

Read Alignment and Filtering

Sequenced reads from ChIP libraries and whole-cell extract (genomic DNA) input controls in the cell types analysed were aligned to the mouse reference genome (MGSCv37/mm9) with Bowtie2 ((Langmead and Salzberg, 2012), bowtie2 -p 10 -x mm9 outputName.sam). The resulting files were further processed using SAMtools to eliminate reads with low mapping scores (i.e. below 40: samtools view -bS -q 40 file.sam > _bestAlignment.bam) as well as PCR duplicates (samtools-0.1.19 rmdup -s _bestAlignment.ba _filtered.bam).

Data Processing for Visualization

Alignment (bam) files with filtered reads were converted to bed format using bedtools (bedtools bamtobed -i _filtered.bam > _filtered.bed). Bed files were further processed in R using the Bioconductor packages GenomicRanges and chipseq. Fragment lengths were estimated for each chromosome using the estimate.mean.fraglen function from the package chipseq, and reads were extended by the median of fragment lengths (extended read files for each library). The genome was then subdivided into consecutive 200 bp bins, and reads in each bin were counted with the countOverlaps function in the GenomicRanges package. Data tracks were normalized by dividing ChIP signal in 200 bp bins by the sample size factors estimated with the DESeq2 package (Love et al., 2014).

Peak Calling

MACS v1.4 (Zhang et al., 2008) was used to call peaks from CTCF, Rad21, H3K4me3 and H3K27ac ChIP-seq data (macs14 -t chipseq.bam -c input.bam -f BAM -g mm –nomodel -n peaks). We used genomic DNA input libraries derived from ES cells. Following manual inspection to confirm the quality of peak calls, final peak lists were obtained after merging the bam files from biological replicates in each condition. In the case of H3K4me1, MACS2 was used to detect enriched loci (macs2 callpeak -t chipseq.bed -c input.bed -f BED -g mm –broad –broad-cutoff 0.1 -n H3K4me1_peaks).

Dynamics of CTCF and Rad21 Binding

We considered a list of peaks identified in either ES (FBS/LIF), NS or both cell types (N= 68,210, hereafter denoted all CTCF peaks). Genomic coordinates of all CTCF peaks were considered, and reads from extended files (see Data processing for visualization) were counted in these intervals. A count table whereby columns corresponded to biological samples and rows to CTCF peak intervals was generated. We applied DESeq2 to quantitatively compare CTCF and Rad21 ChIP-seq signals (Figure 3B). Normalized LFC of ChIP-seq signal were obtained from DESeq2 objects using the results function and considered in Figures 3B, 3C, and S3. The two classes of CTCF peaks were defined as described in Identification of contact domains.

Dynamics and CTCF Directionality

Genome-wide annotation of CTCF motifs was obtained using FIMO from the MEME analysis suite (Grant et al., 2011). Motifs with p-values $<1x10^{-4}$ were considered. In a case where multiple motifs were identified for a peak, that with the lowest p-value was considered. CTCF signal was computed and normalized as described in Dynamics of CTCF and Rad21 binding. Peaks with LFC>0 (NS/ES(FBS/LIF)) were deemed increased in NS cells. Peaks were then stratified according to whether a CTCF motif was found in the positive (forward peaks) or negative (reverse peaks) strand. Genomic intervals defined by the midpoints of loop anchors identified as increased in NS cells (NS loop domains; ES (FBS/LIF) vs NS cells) were considered. Each NS loop domain was divided into 250 non-overlapping tiles of equal size. Thus, for each loop we obtained tile size (TS) = loop size (bp) / 250. Ten tiles of size TS were appended to the loop start and end. The overlap with CTCF peak classes was computed for each of the 270 tiles. This facilitated rescaling of NS loop domains to compare the distribution of CTCF peaks.

Assignment and Classification of Promoter Regions

Genome annotation was obtained from Ensembl. We considered putative promoter regions from -100 to + 100 bp around transcription start sites of protein coding genes. Promoters were deemed active if they overlapped an H3K4me3 peak. Those that did not overlap an H3K4me3 peak were considered inactive.

Identification of Enhancers from ChIP-Seq Data

Peaks from H3K4me1, H3K4me3 and H3K27ac ChIP libraries were considered. Putative enhancers were identified as H3K4me1 peaks that did not overlap either an H3K4me3 peak or a defined promoter region (Ensembl annotation). Enhancers were considered active if the H3K4me1 peak overlapped an H3K27ac peak. Those that did not overlap an H3K27ac peak were deemed poised. Induced enhancers were defined as the subset determined to be enhancers active in NS cells that did not overlap those active in ES cells. Repressed enhancers were defined as enhancers active in ES cells that did not overlap enhancers active in NS cells.

Calculation of Domain-Wise Coverage of H3K4me1 Marks

Data from H3K4me1 ChIP-seq and corresponding input samples were processed using the BinarizeBed function from the chromHMM tool (Ernst and Kellis, 2012) to evaluate genomic regions spanning 200 bp bins for significant H3K4me1 enrichment. Coverage of H3K4me1-enriched bins was then computed for each TAD.

Genomic Features and Loop Anchors

To compute intersection with CTCF binding sites (Figures S1D and S2A), we extended loop anchor coordinates by 10kb in both directions. For induced and reduced loops identified from in-situ Hi-C data through comparative analyses, the minimal size of a loop anchor was 5 kb (single bin) ranging to several tens of kilobases after the clustering/merging described above. For relating to CTCF and other functional elements in the genome, we considered the midpoint of each anchor and extended it by 5 kb in both directions.

Gene Ontology Analysis

To identify genes demarcated by the anchors of dynamic loops, we considered the genomic intervals between the loop anchors of induced and repressed loops (ES cells (FBS/LIF) versus NS cells; similar enrichments were obtained with data from 2i/LIF cultures). We annotated protein coding genes related to induced or repressed loops based on the overlap of putative promoter regions. Rare cases of redundancy were observed (e.g., nested loops), whereby one of the loop anchors is common to the repressed and induced sets. Genes found within both groups were removed from consideration. Gene sets were analysed using the updated version of DAVID (Huang et al., 2009) (david.ncifcrf.gov, version 6.8).

RNA-Seq Data Analysis

Reads were aligned to the mouse reference genome (MGSCv37/mm9) using Tophat2 (Kim et al., 2013). Reads with alignment quality score below 10 were removed. For each gene the number of mapped reads placed within the CDS (spanning all annotated exons) were counted with HTSeq (Anders et al., 2015) and used as input for differential expression analysis with DESeq (Anders and Huber, 2010).

Enrichment of Neuronal Genes and Enhancers within Induced Loop Domains

Affymetrix microarray expression profiles of 75 different tissues (Su et al., 2004) and in Neuron Progenitor (NP) cells (Mikkelsen et al., 2007), were obtained from the Gene Expression Omnibus (GEO, Key Resources Table) and normalized by variance stabilization in the vsn package from Bioconductor (Huber et al., 2003). Only “perfect match” probes were retained for further analysis and the expression values from each probeset were summarized with the median-polish method (Irizarry et al., 2003) implemented in the affy package from Bioconductor. We annotated probesets to genes; in cases where multiple probesets targeted a single locus, we chose the probeset displaying the highest variability in signal across all tissues.

Neural genes were defined those highly expressed (within the top 15% of genome-wide expression levels) in at least one adult neuronal tissue for which data were available ("cerebellum", "cerebral cortex", "cerebral cortex prefrontal", "dorsal striatum", "hippocampus", "hypothalamus", "microglia", "nucleus accumbens", "olfactory bulb", "pituitary", "spinal cord") and that were not upregulated in NS cells relative to ES cells.

We incorporated enhancer elements annotated in cerebellum, cortex and whole brain (E14.5) samples (Shen et al., 2012) (chromosome.sdsc.edu/mouse/download.html).

We identified induced loop domains that did not overlap lost domains, as well as the inverse. We then computed enrichment of neuronal genes and enhancers in the filtered sets of induced and lost loop domains. For each of the two sets, we enumerated loops that contained at least one neuronal gene and did not contain any gene induced in NS cells (adjusted $p<0.01$, $\log_2(NS/ES)>1.5$, DESeq method). We then related this to the number of loops that did not contain any neuronal gene nor a gene upregulated in NS cells, but for which at least one gene was included in the GNF1M microarray design (Su et al., 2004). Enrichment between the two groups was compared with Fisher’s exact test.

Dynamics of Intra-Domain Promoter-Enhancer Contacts

We considered induced, common and reduced loop domain coordinates. We removed induced loops that overlapped reduced or common loops. We also removed reduced loops that overlapped either induced or common loops. We shifted the starts and ends of loop domain coordinates by 20kb in the 3′ and 5′ direction, respectively, to remove loop anchors from consideration. We also removed intra-domain interactions spanning less than 20 kb. We then identified bin pairs at 5kb resolution that spanned active promoters (based on the overlap with H3K4me3) and active enhancers. To increase the stringency of this comparison, for each domain we computed the average ratio of contacts derived from in-situ Hi-C data between promoters and enhancers in ES and NS cells. Figure 6E (boxplot) displays the distribution of these values.

DATA AND SOFTWARE AVAILABILITY

Sequencing data are available through the ArrayExpress repository under accessions E-MTAB-2125, E-MTAB-2063, E-MTAB-5732, E-MTAB-6591. Analysis code and processed data can be obtained from www-huber.embl.de/projects/stemcell3Dloops.

Cell Systems, Volume 7

Supplemental Information

Gain of CTCF-Anchored Chromatin Loops

Marks the Exit from Naive Pluripotency

Aleksandra Pękowska, Bernd Klaus, Wanqing Xiang, Jacqueline Severino, Nathalie Daigle, Felix A. Klein, Małgorzata Oleś, Rafael Casellas, Jan Ellenberg, Lars M. Steinmetz, Paul Bertone, and Wolfgang Huber

Supplementary Table 1, related to Figure 1 | Chromatin conformation data produced for this study.

Sample	Protocol	Number of sequenced reads	Uniquely aligned read pairs	Final contacts (MAPQ≥30)	Intra-chromosomal (%)	Inter-chromosomal (%)
**ESC (FBS/LIF) rep1**	TCC	134,434,660	81,290,648	53,023,831	89.1	10.85
**ESC (FBS/LIF) rep2**	TCC	171,193,881	105,990,730	69,261,153	89.3	10.6
**NSC rep1**	TCC	127,309,593	94,140,986	64,491,743	89.6	10.4
**NSC rep2**	TCC	171,338,684	85,238,722	49,202,635	87.2	12.8
**ESC (2i/LIF) rep1**	TCC	120,729,477	93,663,961	62,710,291	90.1	9.9
**ESC (2i/LIF) rep2**	TCC	146,159,495	110,767,469	71,398,936	88.2	11.8
**EpiSC rep1**	TCC	72,946,843	47,857,076	32,824,806	85.7	14.3
**EpiSC rep2**	TCC	266,948,104	177,889,434	113,867,789	87.8	12.2
**ESC (FBS/LIF) rep1**	*In situ* Hi-C	611,626,283	561,290,686	399,655,481	87.6	12.4
**ESC (FBS/LIF) rep2**	*In situ* Hi-C	622,966,917	572,610,548	408,970,163	83.5	16.5
**NSC rep1**	*In situ* Hi-C	657,535,758	604,637,569	430,767,343	81.3	18.7
**NSC rep2**	*In situ* Hi-C	619,104,144	563,686,394	398,857,642	80.9	19.1

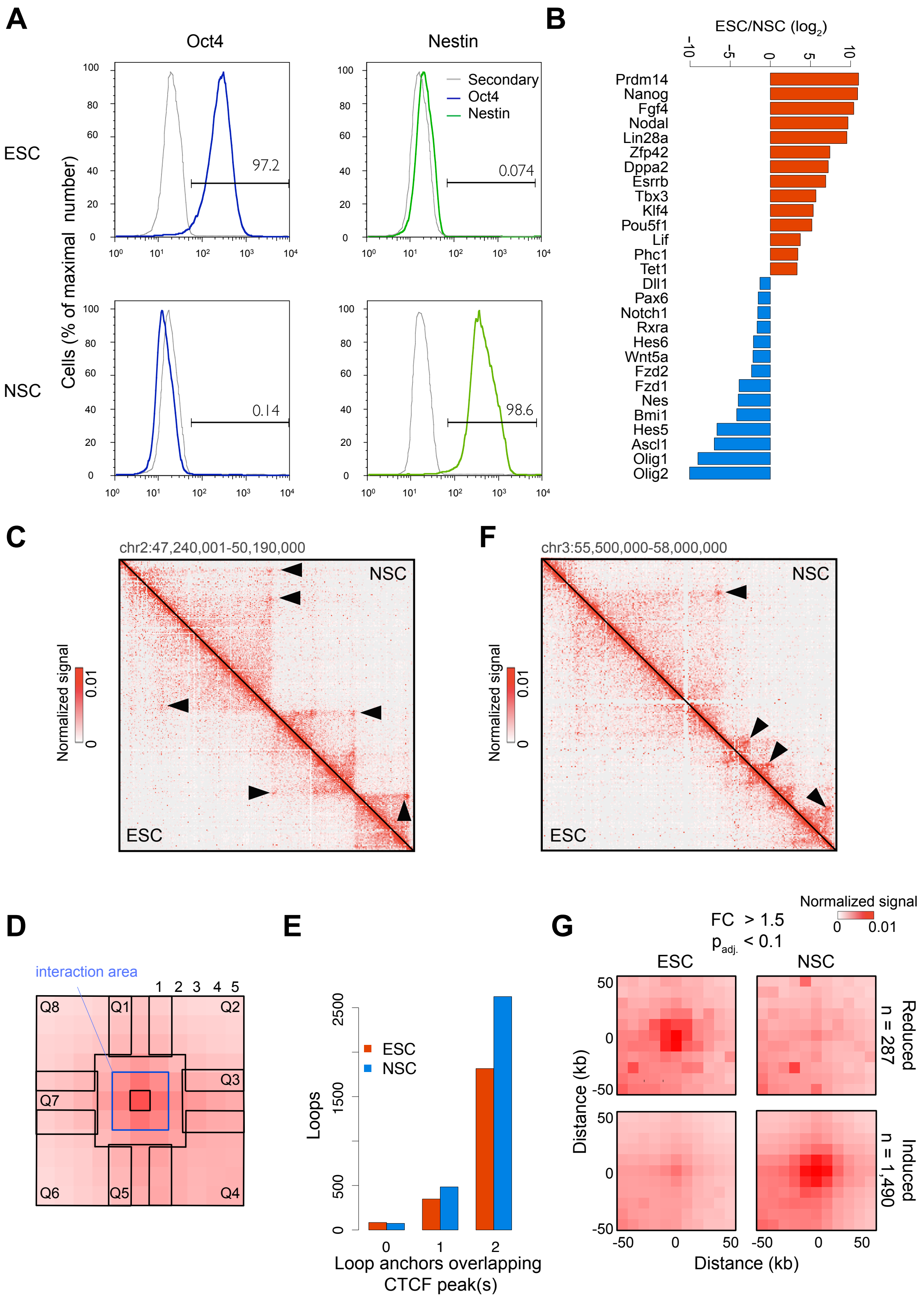

Figure S1, related to Figure 1 | Loops are primarily gained upon differentiation. A) Flow cytometry analysis of Oct4 and Nestin in ESCs and NSCs. B) Comparative expression of key pluripotency (red) and neural stem cell-related genes (blue) from RNA-seq data (adjusted p<0.0001, *DESeq* method). C) Example of chromatin loops in ESCs and NSCs (TCC data). D) Approach for the identification of loops. Bin pairs (pixels) in the area -50 to + 50 kb around each interacting 10 kb bin pair were analyzed for the presence of interactions. Eight overlapping areas of varying shape were considered (Q1 to Q8, green and white areas in the schematic; note that some of these overlap others). The number of significant interactions in the pixels immediately surrounding and including the center pixel (interaction area) was counted. Loops were defined as pixels where this number was at least two in the interaction area, and where at most two interactions were observed in each of areas Q1 to Q8. E) The majority of loops identified from TCC data connect anchors overlapping CTCF binding sites. F) Example of loops induced in NSCs. G) Composite profile of normalized TCC signal from reduced (top) and induced (bottom) loops in ESCs (left) and NSCs (right) cells (Wald test, adjusted p=0.1 and FC>1.5).

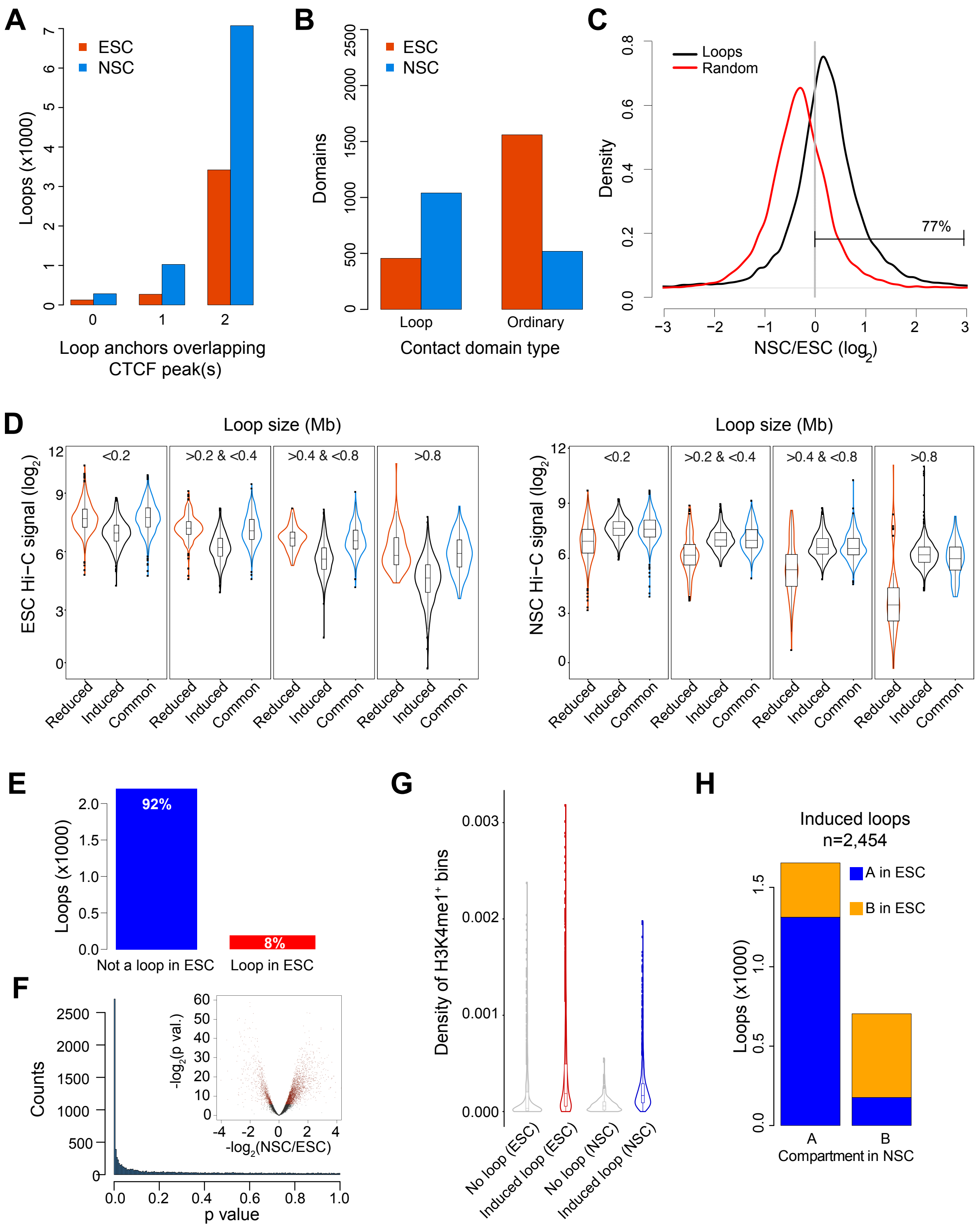

Figure S2, related to Figure 1 and Figure 2 | High resolution in-situ Hi-C reveals gain of loops upon differentiation. A) Loops increase upon ESC differentiation to NSCs. The majority of loops connect loci overlapping CTCF binding sites. B) The ratio of loops to ordinary domains increases upon differentiation. C) Loop signal is primarily increased over random (*in situ* Hi-C data). Normalized loop signal in ESCs and NSCs was considered (STAR Methods). D) Loops defined as dynamic via *DESeq2*-based analysis (Wald test) display signals comparable to common loops in the condition where they are identified as significantly stronger (*in situ* Hi-C data). E) Loops induced upon differentiation are largely undetectable in ESC. Only 8% of loops displaying signals stronger in NSCs (Wald test, FDR=0.05 and FC>1.5) are identified in ESCs (*HiCUPPS* method). F) Distribution of p-values from comparison of loop signals in ESC and NSC (Wald test, *in situ* Hi-C data). Inset: volcano plot depicting the relationship between $\log_{10}$ p values to $\log_2$ fold change of loop signals. G) Loops are formed in regions enriched for H3K4me1. Induced loop domains are already established in open chromatin in ESCs. H) Genomic intervals defined by the anchors of loops induced upon ESC differentiation to NSCs are within the euchromatic compartment (A) in both cell types. Compartmental assignment was evaluated genome-wide at 50 kb resolution. Loops were assigned to compartments based on annotation of the majority of bins mapping within the loop interval.

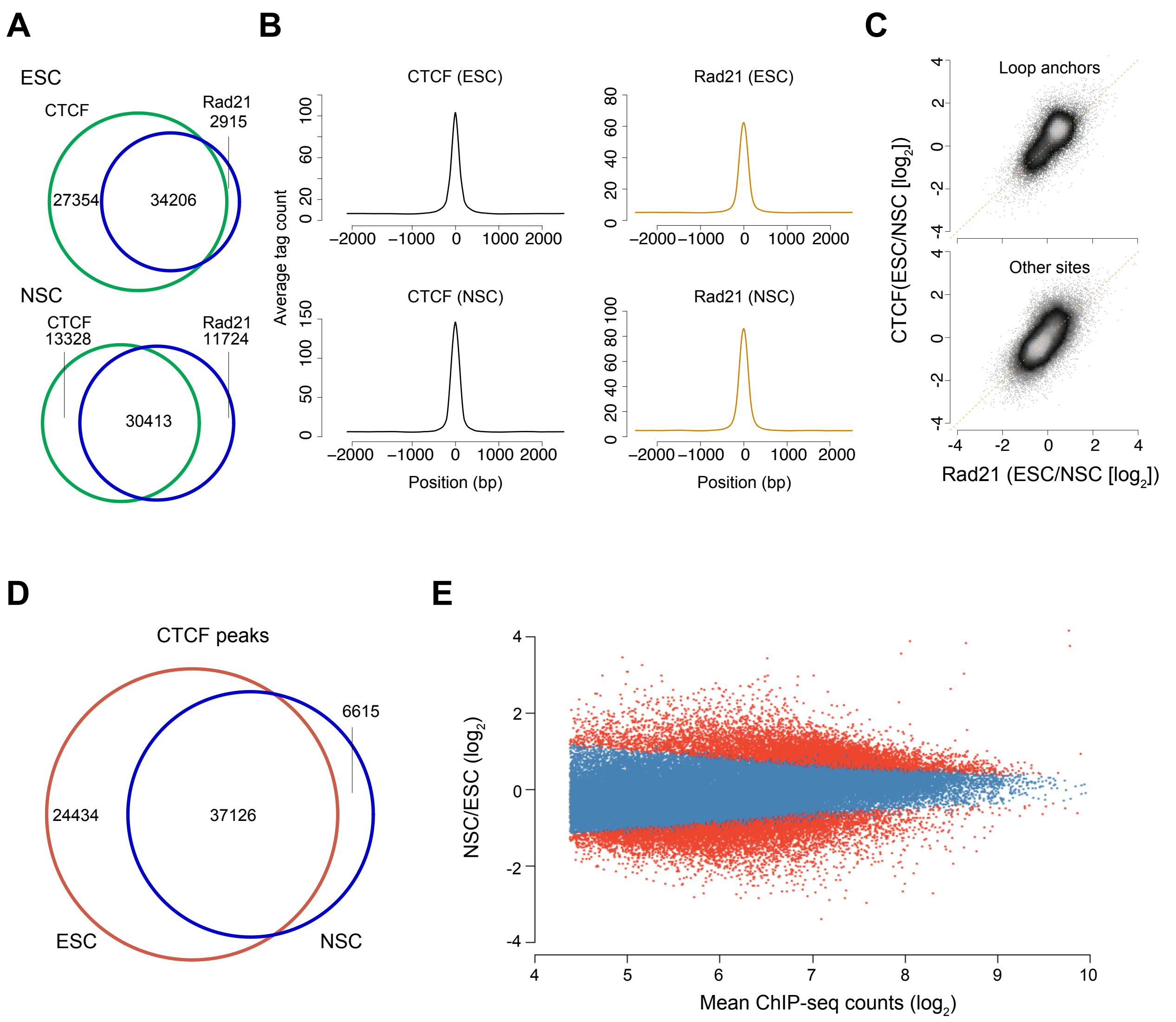

Figure S3, related to Figure 3 | CTCF and Rad21 following ESC differentiation to NSC. A) Overlap between the genomic positions of CTCF and Rad21 peaks identified in ESCs (top) or NSCs (bottom). B) Average profile of CTCF and Rad21 ChIP-seq fragments around the summits of CTCF peaks in ESCs and NSCs (top and bottom panels, respectively). C) Variation in CTCF and Rad21 signals in ESCs and NSCs is highly correlated both at loop anchors (*in situ* Hi-C data) and at other CTCF sites throughout the genome. *DESeq2*-normalized ChIP-seq signals in ESCs and NSCs were considered. D) Comparison of CTCF peaks identified in ESCs and NSCs. E) Log_2 fold change (NSC/ESC) of CTCF ChIP-seq signal in the function of the average signal strength (MA plot). Red: peaks with significant changes in CTCF binding (adjusted p<0.1).

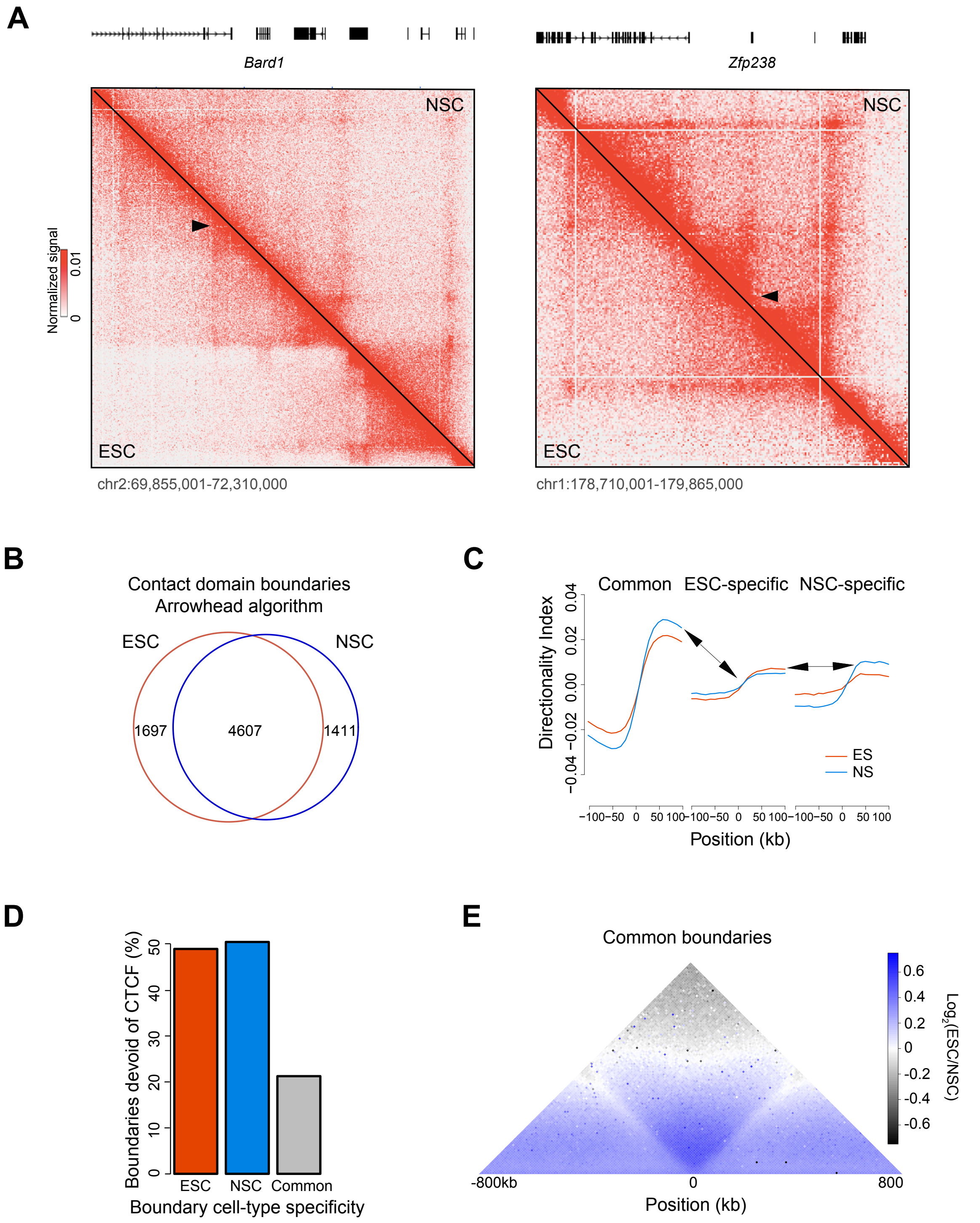

Figure S4, related to Figure 4 | Contact domain boundaries are weaker in ESC than in NSC. A) Cell type-specific contact domain boundaries. Left: ESC-specific contact domain boundary at the 3' end of the transcriptionally down-regulated *Bard1* locus (adjusted p<0.1, $\log_2$(ESC/NSC)>1.5, *DESeq* method). Right: NSC-specific contact domain boundary at the 5' end of transcriptionally upregulated *Zfp238* (adjusted p<0.1, $\log_2$(ESC/NSC)<-1.5, *DESeq* method). B) The majority of boundaries are common to both cell types. Shared and unique domain boundaries identified in ESCs and NSCs. C) Average profiles of the directionality index around common, ESC- and NSC-specific contact domain boundaries. Cell type-specific boundaries are weaker than shared instances. Overall, boundaries are stronger in NSCs than in ESCs. D) Cell type-specific boundaries are more frequently devoid of CTCF than common ones. E) Composite profile of normalized *in situ* Hi-C contacts around common boundaries of contact domains. ESCs display elevated numbers of Hi-C contacts crossing common domain boundaries.

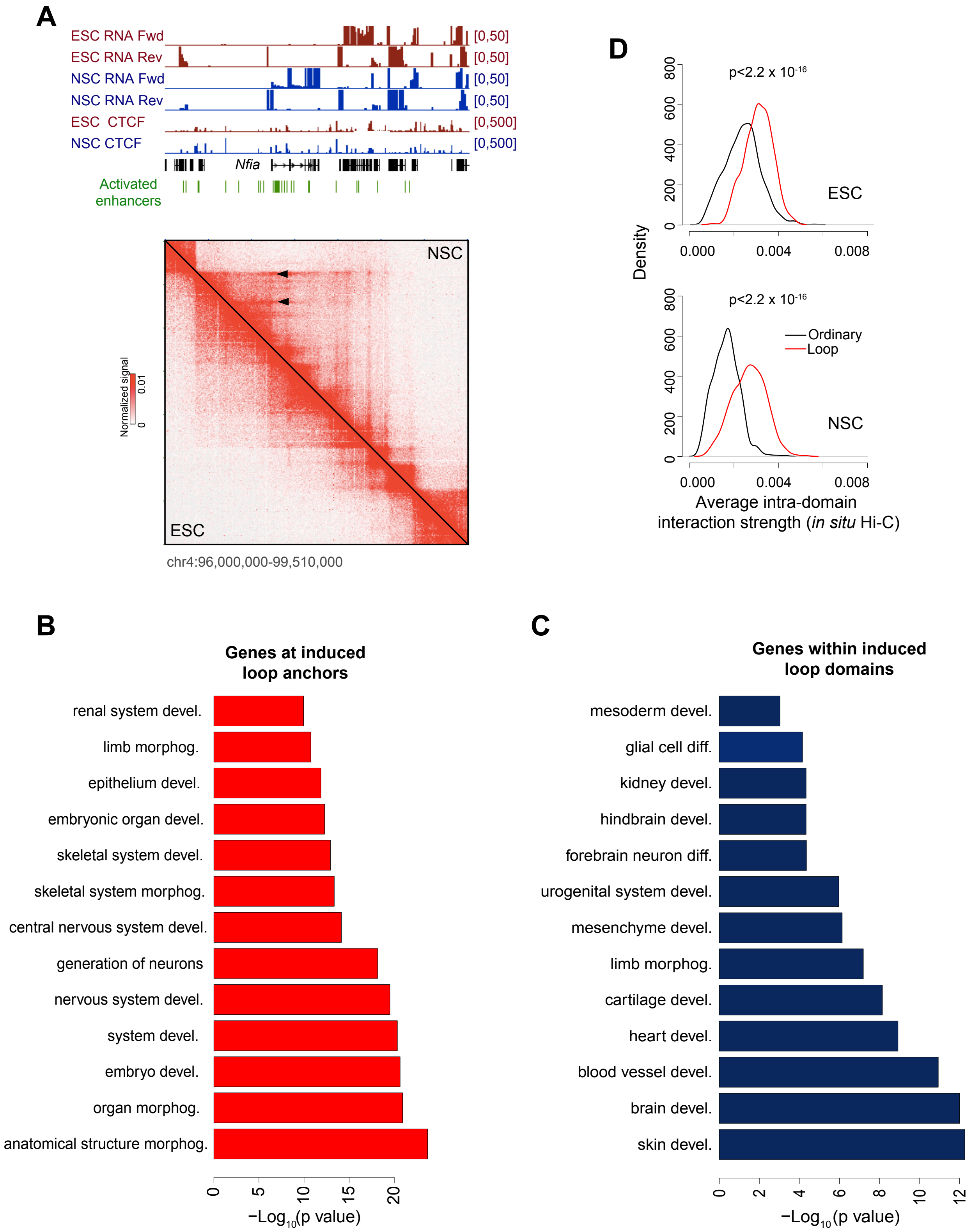

Figure S5, related to Figure 6 | Loop induction is associated with developmental control of gene expression. A) Induction of loops connecting the promoter of *Nfia* with distal regulatory elements in NSCs compared to ESCs. B) Gene Ontology (GO) analysis of genes with promoter regions intersecting anchors of induced loops. GO terms associated with developmental processes are displayed (Benjamini-Hochberg corrected p values). C) Genes within genomic intervals defined by the anchors of induced loops are implicated in development. Analysis as in panel B. D) Loop domains display stronger interactions on average than ordinary contact domains (two-sided t test). Interactions between loci located closer than 20kb were not considered in this analysis.

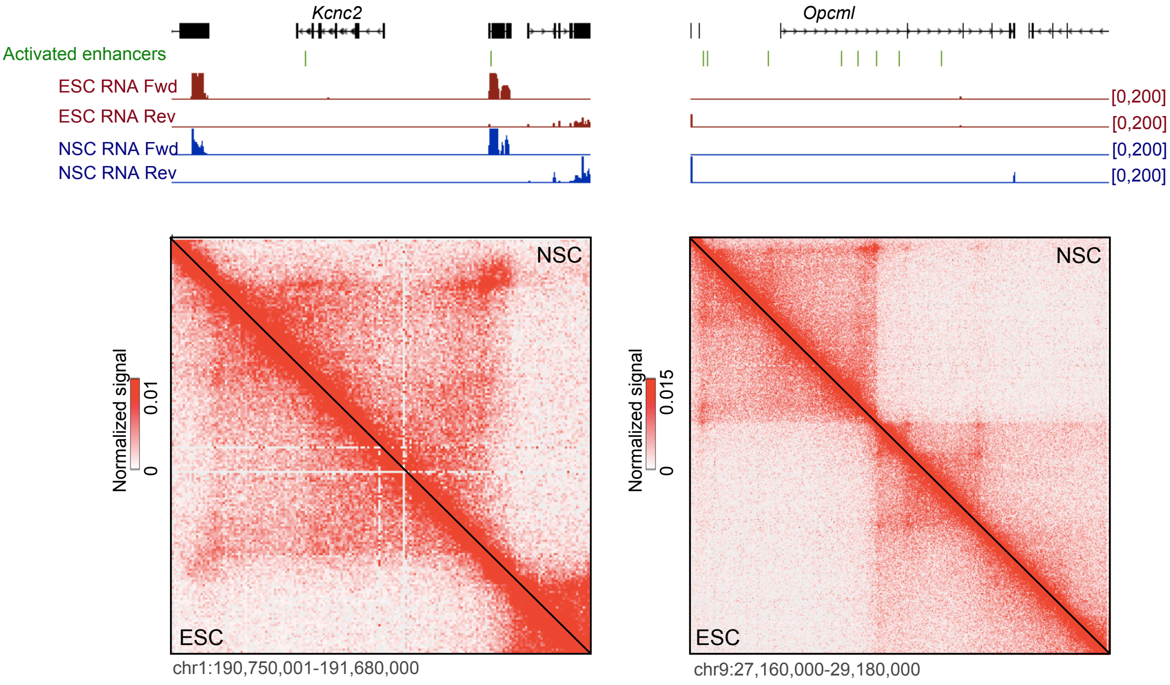

Figure S6, related to Figure 6 | Loops around genes expressed in adult neuronal tissues are induced in NSCs. Induced loops around *Kcnc2* (right), and *Opcml* (left), neural genes not yet induced in NSCs (*in situ* Hi-C data).

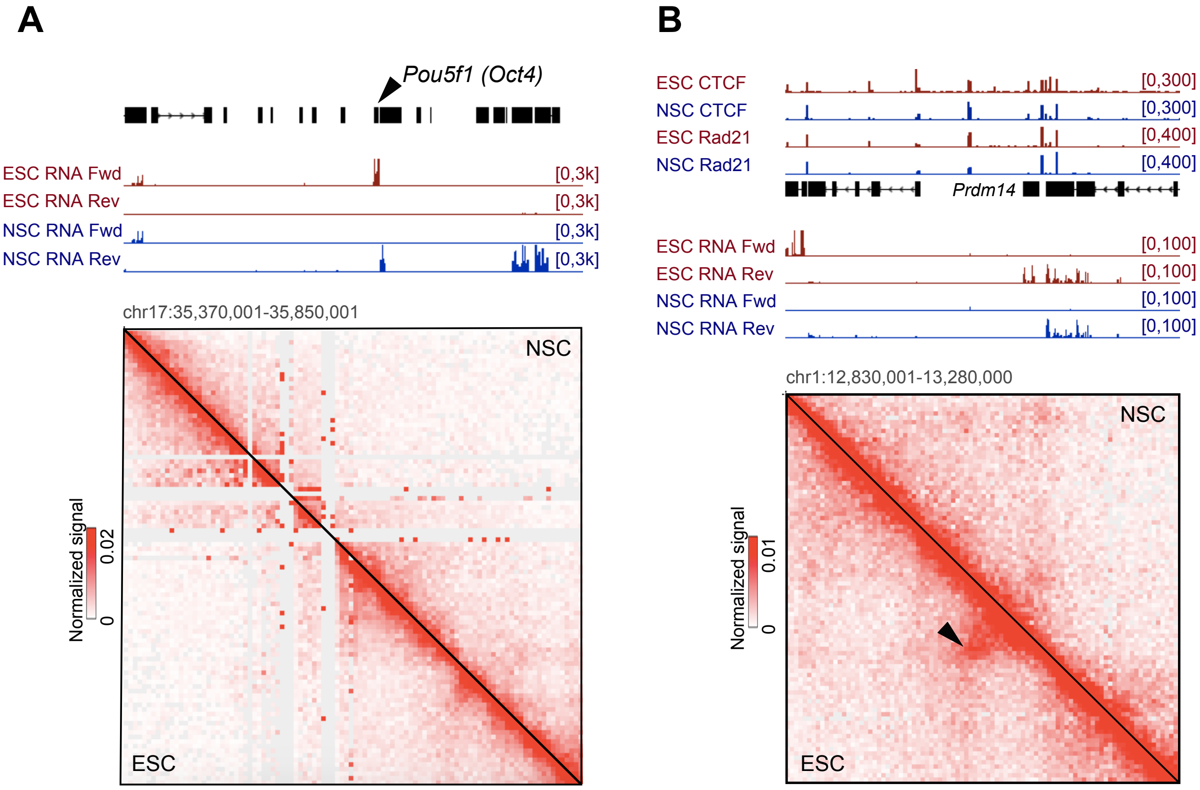

Figure S7, related to Figure 6 | Loop dynamics around pluripotency related genes. A) Absence of long-range loops around the *Pou5f1* (*Oct4*) locus in ESCs and NSCs. B) Short-range loop involving the promoter of pluripotency-associated gene *Prdm14*. This structure is present in ESCs and significantly diminished in NSCs ($p<10^{-4}$, $\log_2$(NSC/ESC) = -1.16). *Prdm14* is downregulated upon exit from pluripotency ($p<10^{-10}$, *DESeq* method, Figure S1B).
